# The prince and the pauper. A tale of anticancer targeted agents

**DOI:** 10.1186/1476-4598-7-82

**Published:** 2008-10-23

**Authors:** Alfonso Dueñas-González, Patricia García-López, Luis Alonso Herrera, Jose Luis Medina-Franco, Aurora González-Fierro, Myrna Candelaria

**Affiliations:** 1 Unidad de Investigacion Biomédica en Cáncer, Instituto de Investigaciones Biomedicas, UNAM/Instituto Nacional de Cancerologia, Mexico City, Mexico; 2 Torrey Pines Institute for Molecular Studies. 5775 Old Dixie Highway, Fort Pierce, Florida 34946, USA; 3 Dirección de Investigación, Unidad de Investigacion Biomédica en Cáncer, Av. San Fernando 22, Tlalpan, 14080 México, D.F., México

## Abstract

Cancer rates are set to increase at an alarming rate, from 10 million new cases globally in 2000 to 15 million in 2020. Regarding the pharmacological treatment of cancer, we currently are in the interphase of two treatment eras. The so-called pregenomic therapy which names the traditional cancer drugs, mainly cytotoxic drug types, and post-genomic era-type drugs referring to rationally-based designed. Although there are successful examples of this newer drug discovery approach, most target-specific agents only provide small gains in symptom control and/or survival, whereas others have consistently failed in the clinical testing. There is however, a characteristic shared by these agents: -their high cost-. This is expected as drug discovery and development is generally carried out within the commercial rather than the academic realm. Given the extraordinarily high therapeutic drug discovery-associated costs and risks, it is highly unlikely that any single public-sector research group will see a novel chemical "probe" become a "drug". An alternative drug development strategy is the exploitation of established drugs that have already been approved for treatment of non-cancerous diseases and whose cancer target has already been discovered. This strategy is also denominated drug *repositioning, drug repurposing*, or *indication switch*. Although traditionally development of these drugs was unlikely to be pursued by *Big Pharma *due to their limited commercial value, biopharmaceutical companies attempting to increase productivity at present are pursuing drug *repositioning*. More and more companies are scanning the existing pharmacopoeia for repositioning candidates, and the number of repositioning success stories is increasing. Here we provide noteworthy examples of known drugs whose potential anticancer activities have been highlighted, to encourage further research on these known drugs as a means to foster their translation into clinical trials utilizing the more limited public-sector resources. If these drug types eventually result in being effective, it follows that they could be much more affordable for patients with cancer; therefore, their contribution in terms of reducing cancer mortality at the global level would be greater.

## Background

At present, cancer remains a significant health problem worldwide. According to International Agency for Research on Cancer-World Health Organization (IARC-WHO) estimates, cancer rates are set to increase at an alarming rate, from 10 million new cases globally in 2000 to 15 million in 2020 [1]. Cancer statistics from the U.S. show a total of 1,368,030 new cancer cases and 563,700 deaths expected; paradoxically, there has been a decrease or stabilization in mortality rates from cancer, particularly in major cancers such as lung, colorectal, prostate, and breast. A recent estimate of trends in 5- and 10-year relative survival of cancer patients in the U.S. in 1998–2003 from the 1973–2003 Surveillance, Epidemiology, and End Results Program data base indicated significant improvements in 5- and 10-year relative survival for 14 of 24 assessed common forms of cancer, such as prostate, breast, and colorectal cancer. Improvements in long-term survival were strongest for prostate cancer, non-Hodgkin lymphoma, and kidney cancer. In general, these improvements are likely the result of progress in early detection, treatment, or both, depending on tumor type [2].

With regard to cancer treatment with drugs, we are currently in the interphase of two treatment eras. So-called pregenomic therapy names the traditional cancer drugs, mainly cytotoxic drug types. This tagging stems from the fact that in general terms, pregenomic cancer drugs were empirically developed based mainly on their capacity to inhibit cancer growth in experimental systems regardless of their nature and potential mechanism of action. Contrariwise, post-genomic era-type drugs refer to rationally based designed drugs in which the startpoint comprises, first, target identification, second, demonstrating that candidate drugs inhibit this target, and third, proving that cancer growth is affected as a consequence of target inhibition.

Whereas the scientific basis for development of these drug classes is strong, our current level of knowledge on the molecular basis of cancer remains a limitation for this design type. To date, successful examples of this newer drug discovery approach are noteworthy, and just to mention a few we site dramatic results with the use of bcr-abl- and c-kit-targeting agents on chronic granulocytic leukemia and gastrointestinal stromal tumors, the impressive results of Epidermal growth factor receptor (EGFR) inhibitors in a small subset of non-small-cell lung cancer, and the efficacy of targeting HER2 by a monoclonal antibody in approximately 30% of patients with breast cancer whose tumors overexpress this oncoprotein. There are many other examples of these drug classes that are already commercially available for the treatment of cancer; however, these pharmaceuticals only provide, albeit significantly, small gains in symptom control and/or survival, whereas others have consistently failed in the clinical testing stage. This picture of the heterogenous results of so-called targeted therapies with respect to their clinical efficacy underscore that while these efforts must continue, parallel efforts are strongly required in cancer biology research for improved prediction of the target to be approached that offer the highest treatment benefit probability. As previously mentioned, these agents are solely effective in tumor types dependent on the pathways being inhibited. It is readily apparent that the majority of solid tumors are the result of numerous genetic and epigenetic alterations; hence, inhibiting a single cellular pathway may not result in significant therapeutic activity. Design of agents that target a number of pathways will possibly increase the therapeutic effect, but could also increase the risk of treatment-related toxicities [3,4].

While it is obvious that the vast majority of knowledge on cancer biology has been generated by investigators from public and non-profit organizations, drug discovery and development is generally carried out within the commercial rather than the academic realm, given the extraordinarily high therapeutic drug discovery-associated costs and risks. Thus, it is exceedingly unlikely that any single public-sector research group will successfully see a novel chemical "probe" become a "drug".

Classical drug discovery involves target discovery and validation, lead identification by high-throughput screening, and lead optimization by medicinal chemistry. Pre-clinical follow-up evaluation includes analysis in animal models of compound efficacy and pharmacology (Administration, distribution, metabolism, elimination [ADME]) and toxicology, specificity, and drug interaction studies. The high-risk/high-reward aspect of drug discovery comprises a greater issue in the commercial realm in terms of new-compound approval and marketability. Therefore, oncological products are subject to the laws of marketing; hence, the majority of the newer cancer products are simply cost-prohibitive to the vast majority of patients worldwide, which has been widely approached and reviewed [5-7]. This important issue has led researchers at non-profit academic organizations to reflect upon alternatives for cancer drug development [8,9].

An alternative drug development strategy is the exploitation of established drugs that have already been approved for treatment of non-cancerous diseases and whose cancer target has already been discovered. This strategy is also denominated drug *repositioning, drug repurposing*, or *indication switch*. Although traditionally development of these drugs was unlikely to be pursued by Big Pharma due to their limited commercial value, biopharmaceutical companies attempting to increase productivity at present are pursuing drug *repositioning*. More and more companies are scanning the existing pharmacopoeia for repositioning candidates, and the number of repositioning success stories is increasing [10]. The best known example is that of sildenafil (Viagra; Pfizer), which was initially developed as an anti-angina medication but possessed the side effect of producing prolonged penile erections in human volunteers [11].

The major advantage of this approach is that the pharmacokinetic, pharmacodynamic, and toxicity profiles of drugs are in general well known; thus, their rapid translation into clinical phase II and III studies is feasible. On the other hand, from the commercial point of view and despite that *repositioning *is observed as a not-very-rewarding adventure, pharmaceutical companies can exploit a number of strategies to add value to this drug development type, such as inventing novel formulations, dosage forms, drug combinations, or geographic strategies that create new barriers to entry. In addition, intellectual property-type *composition-of-matter *and *use *patents can be granted, as well as marketing exclusivity for different time periods for Federal Drug Administration (FDA) approval of new indications in a pediatric population, for a known compound for a new indication, a new chemical entity, or in an orphan population [10].

The process of *repositioning *and in particular in the cancer therapeutic field is not yet systematized. As shown in this review, clues for cancer activities of the majority of non-cancer drugs presented arose from serendipity and novel insights into the molecular pathology of cancer, for example, the realization that AMP-activated protein kinase (AMPK), the target of metformin is also a cancer target, and so on. Off-target toxicity also serves as a way to discover antitumor effects of known drugs, for instance, the effects on the DNA methylation-autoimmune disease of drugs such as procainamide and hydralazine. Although this approach may be efficient with effective drugs, it is biased and limited to a single drug type.

Recently, O'Connor and Roth [9] proposed an approach more likely to be successful in achieving the ultimate goal of providing new drugs, one in which already available medications – the majority of which are off-patent – are simultaneously screened employing several *in vitro *and *in vivo *model systems. This approach utilizes existing medications that are subsequently used as probes for pre-clinical molecular target- or phenotype-based drug discovery efforts. In contrast, the proposed approach blindly screens existing compounds against a multitude of targets, and therefore identifies either possible therapeutic benefits or side effects in a non-biased fashion.

The objective of this review was to provide noteworthy examples – but not a comprehensive review on each of these – of known drugs whose potential anticancer activities have been highlighted, to encourage further research on these known drugs as a means to foster their translation into clinical trials utilizing the more limited public-sector resources [Table 1]. If these drug types eventually result in being effective, it follows that they could be much more affordable for patients with cancer; therefore, their contribution in terms of reducing cancer mortality at the global level would be greater.

**Table 1:** Summary of noncancer drugs, their primary indication, noncancer and cancer targets

AGENT	PRIMARY INDICATION	ON-TARGET Primary effects	ON-TARGET Antitumor effects	OFF-TARGET Antitumor effects
**CCA**				
Verapamil Diltiazem	Anti-arrhytmic	L-type Ca^2+ ^channels	*L-type Ca*^2+^*channels*	Voltage-gated K+ channels MDR proteins
				
**Inotropics**				
Digitalis	Heart failure	Na(+)/K(+)-ATPase	*Na(+)/K(+)-ATPase*	Death receptors Glycolysis
				
**RAS**				
Losartan	Hypertension	ACE, AT_1_R	*AT*_1_*R*	
Captopril	Heart failure			
				
**Antianginal**				
Nitroglycerin	CHD	GMP	*cGMP*	
				
**Alpha_1_-adrenoceptor antagonists**				
Terazosin	Hypertension	A_1_R	*A*_1_*R*	EGFR
Alfuzosin	BPH			
Prazosin				
				
**Vasodilator**				
Hydralazine	Hypertension	Unknown		DNA methylation
				
**Antiarrhytmic**				
Procainamide	Arrhytmias	Na+ channels		DNMT1
				
**Local anesthetic**				
Procaine	Local anesthesia	Na+ channels		DNA methylation
				
**Antiepileptic**				
Valproic acid	Epilepsia	GABA ergic	*Blocking NMDA Na+ channels*	Class I-II HDACs PPARγ
				
**Antidiabetics**				
Glitazones	Diabetes mellitus	PPARγ	*PPARγ*	
Metformin	Diabetes mellitus	AMPK	*AMPK*	
				
**Antiobesity**				
Orlistat	Obesity	Fatty-Acid Synthase	*Fatty-Acid Synthase*	
				
**Cholesterol lowering agents**				
Statins	Cholesterolemia	HMGC	*HMGC*	
				
**Antimalarial**				
Chloroquine	Malaria	Lysosomas	*Lysosomas*	Autophagia
				
**Abortive**				
Mifepristone	Abortion	Progesterone receptor	*Progesterone receptor*	MDR/MPR

Italic fonts indicate that the targets are shared by the pathological conditions (on-target effects).

Underlined fonts indicate that the antitumor effect is explained by different targets (off-target effects).

This table indicates that some "benign" conditions share molecular alterations with malignant diseases (one target-several indications).

CCA: calcium channel antagonists; RAS: renine-angiotensin-system; CHD: coronary heart disease; BPH: benign prostatic hyperplasia.

### Cardiovascular agents

#### Calcium channel antagonists (CCA) as antihypertensives and antiarrhythmics

The antihypertensive and antianginal effectiveness of CCA have been documented for more than 30 years. Since that time, these agents have enjoyed increasingly widespread use in the management of high blood pressure, angina pectoris, and certain cardiac arrhythmias. Calcium ions (Ca^2+^) are the most important cellular messengers in biology [12]. Ca^2+ ^entry into the mammalian cell cytosol initiates such responses as excitation and contraction. Ca^2+ ^entering human heart cells also regulates pacemaking and atrioventricular conduction, and may influence cell growth and differentiation. Ca^2+ ^enters the cell through plasma membrane channels that are members of a large family of ion channels. The most important Ca^2+ ^channels in the cardiovascular system are the voltage-gated channels, which are opened by changes in membrane potential. There are at least six types of voltage-gated Ca^2+ ^channels, including L-, N-, P-, Q-, R-, and T-type channels. N-, P-, Q-, and R-type channels are located in neurons, while L- and T-types are localized in the cardiovascular system [13]. L-type Ca^2+ ^channels are the most important plasma membrane Ca^2+ ^channels in heart and vascular smooth muscle and bind Ca^2+ ^channel blockers currently used in clinical practice, including dihydropyridines (e.g., nifedipine), phenylalkylamines (e.g., verapamil), and benzothiazepines (e.g., diltiazem).

CCA vary in chemical structure and clinical effects. All commercially available agents target the L-type channel. Diltiazem and verapamil are non-selective agents, and both at equivalent concentrations cause vasodilation, depress cardiac contractility, and inhibit atrioventricular conduction, this in contrast to predominant vasodilation exerted by dihydropyridines (nifedipine and amlodipine). The therapeutic uses of these agents rely on their chemical structure and cardiovascular profile. Diltiazem and verapamil are effective in angina and high blood pressure, as well as against certain cardiac arrhythmias due to their ability to inhibit atrioventricular conduction [14,15].

#### Calcium channel antagonists as anticancer agents

Calcium is recognized as an important regulator of many essential cellular functions, and in the majority of proliferating cells calcium acts as a general mitogen to stimulate growth. Other mitogenic effect-associated second messengers include generated phospholipids and diacylglycerol. It has been shown that in the presence of diacylglycerol, protein kinase C is activated by a rise in cytosolic-free calcium [16,17]. Once activated, protein kinase C isoenzymes catalyze the phosphorylation of a number of cellular proteins necessary for proliferation [16-18]. In addition, transient rises in cytosolic calcium have shown to initiate activation of the calcium receptor calmodulin, which may also play an important role in the regulation of proliferation [16]. Tumors are generally recognized as possessing unusually high calcium levels. It has been suggested that the high calcium level is due to either excessive influx of extracellular calcium or the ability of neoplastic mitochondria to retain higher calcium concentrations [19]. It is plausible that high intracellular calcium levels yield increased calcium second-messenger system activation [19]. CCA have demonstrated to induce apoptosis and decrease cellular proliferation in many cancer cell lines *in vitro *and *in vivo *by a yet undefined mechanism that may or not depend on blocking any ionic channels including L-type channels, because in many studied systems cells do not express voltage-operated calcium channels, nor has inhibition of calcium-dependent, secondary-messenger system inhibition been demonstrated consistently [20-27]. Possible mechanisms of growth inhibition by CCA include interference with the action of protein kinase C, calmodulin, and phosphodiesterase, or the c-ras oncogene guanosine triphosphate-binding protein [28]. CCA also increase cytotoxicity when added to chemotherapy, an effect attributed to blocking the multidrug resistance protein P-glycoprotein, which acts as an adenosine triphosphate-dependent drug efflux pump, reducing intracellular chemotherapeutic drug accumulation [29].

The first clinical testing of CCA against cancer exploited their anti-mdr action for increasing sensitivity to cytotoxic anticancer drugs. In a prospective study in 99 patients with anthracycline-resistant metastatic breast carcinoma randomized to vindesine-5FU with or without verapamil, treatment was well tolerated and no verapamil-attributed side effects were detected. Response and survival were statistically superior in patients receiving verapamil [30]. Increased responses and survival were also observed in a trial performed in 72 patients with non-small-cell lung cancer randomized to vindesine-ifosfamide-mesna plus minus verapamil [31]. However, a phase III randomized study of vincristine, doxorubicin, and dexametasone (VAD) against the same regimen plus oral verapamil in patients with refractory myeloma reported in 1995 failed to show a survival advantage. Response rates were similar, with an overall response rate of 41% for the VAD-alone arm and 36% for the VAD/v arm. Overall survival of patients was also similar, with median survival of 10 months for the VAD arm and 13 months for the VAD/v arm [32]. The results of this trial discouraged clinical investigation of CCA in further phase III trials. However, an important question remaining comprises whether this apparent lack of efficacy is due to that the trial was underpowered. For the sake of placing this trial into perspective, the approval of bortezomib for refractory multiple myeloma was based on a comparison against high-dose dexametasone in 669 patients [33].

Current research efforts concerning CCA in cancer are focused on meningioma. Diltiazem, verapamil, and nifedipine have shown to induce growth inhibition in meningioma cell cultures, as well as in a mouse xenograft model [34-38]. In addition, diltiazem and verapamil added to HU or RU486 increase meningioma growth inhibition *in vitro *by inducing apoptosis and G1 cell-cycle arrest and *in vivo *by affecting microvascular density [39]. On this basis, a clinical trial program of verapamil alone or with hydroxyurea as treatment for recurrent or refractory meningioma is ongoing [40].

#### Digitalics as inotropics for heart failure

Positive inotropic agents are employed to improve the impaired cardiac contractility that characterizes chronic heart failure, and digitalics are the traditional drugs administered for this purpose. The most commonly used preparation of digitalis is digoxin, obtained from the leaves of *Digitalis lanata*, a common flowering plant known as foxglove. Digitalis inhibits active sodium and potassium transport across cell membranes by specific-site binding to the extracytoplasmic surface of the sodium- and potassium-activated adenosine triphosphatase (NaK ATPase) alpha subunit pump; this binding is a reversible process. The net result is an increase in intracellular sodium and calcium concentrations and a decrease in intracellular potassium concentration. Digitalis increases phase 4 of the action potential in the majority of myocardial tissue, leading to a reduction of conduction velocity with increased automaticity and ectopic activity. Improved inotropy is due to increased cytosolic calcium-ion concentration during systole. Digitalis additionally possesses a negative chronotropic action that is partly a vagal effect and partly a direct effect on the sinoatrial node [41-43].

The therapeutic daily dose of digoxin ranges from 0.005 mg/kg in premature infants to as much as 0.75 mg in adults. Digoxin tablet absorption is 70–80%, while its bioavailability is 95%. The kidney excretes 60–80% of the digoxin dose unchanged. Onset of action via oral administration occurs in 30–120 minutes; onset of action with intravenous (i.v.) administration occurs in 5–30 minutes. Peak effect with PO dosing is 2–6 hours, and that with i.v. dosing is 5–30 hours. Only 1% of the total amount of digoxin in the body is in the serum; of this amount, approximately 25% is protein bound. Volume of distribution is 6–10 L/kg in adults, 10 L/kg in neonates, and as much as 16 L/kg in infants and toddlers. At therapeutic levels, elimination half-life is 36 hours with renal excretion. In acute digoxin intoxication in toddlers and children, average plasma half-life is 11 hours. With acute intoxication, time zero-extrapolated plasma concentrations are lower in toddlers than in infants and older children due to their increased distribution and clearance volumes [44,45].

The lethal dose of digoxin is considered as 20–50 times the maintenance dose taken at once. In healthy adults, a dose of < 5 mg seldom causes severe toxicity, but a dose of > 10 mg is nearly always fatal. In pediatric population, ingestion of > 4 mg or 0.3 mg/kg portends serious toxicity. Although digitalis-intoxication incidence and severity is decreasing, surveillance of this important complication of therapy is essential. Digoxin-interacting drugs are numerous and include amiodarone, propafenone, quinidine, verapamil, nifedipine, diltiazem, levothyroxine, cyclosporine, flecainide, disopyramide, omeprazole, tetracycline, and erythromycin. These agents affect digoxin clearance or absorption, thus necessitating digoxin-dose alteration in patients taking these medications. Furthermore, patients with renal insufficiency may require a downward-adjusted digoxin dose to avoid digitalis intoxication [46,47].

Numerous studies confirm that digoxin does not prolong survival in patients with systolic heart failure, but the drug is associated with reduced hospital admissions, improved functional class, reduced symptoms of heart failure, and improved quality of life. Digoxin is also an effective agent against atrial tachyarrhythmias at rest in patients with left ventricular dysfunction, but exhibits limited efficacy in controlling ventricular atrial-arrhythmia rate during exertion [48,49].

#### Digitalics as anticancer agents

Accumulating pre-clinical and clinical data suggests that digitalic drugs might be used in cancer therapy. Early observations reported that patients with breast cancer receiving digitalis had tumor cells with more benign characteristics than tumor cells in patients not receiving this cardiac glycoside, as well as an apparent lower recurrence rate [50].

Recent reports have shown that ouabain and related digitalics can inhibit growth and induce apoptosis in human cancer cells in culture and xenografted in immunodeficient mice at concentrations commonly found in the plasma of cardiac patients treated with this drug [51]. These effects are highly selective for human cells and depend on Na(+)/K(+)-ATPase inhibition, because studies on [3H] ouabain binding demonstrate that, in comparison with human cell lines, no significant binding of the drug is observed in mouse- and Chinese hamster-derived cells, which are resistant to the antiproliferative effects of these drugs. Thus, Na+/K+ ATPase from cells of the resistant species is inhibited at much higher concentrations of ouabain and digitoxin in comparison with the human cell enzyme, and good correlation is observed between these concentrations and those reported for enzyme inhibition from isolated heart muscles of the same species [52].

The physiological effects of digitalis on blood pressure and cardiac activity are consistent with an Na(+)-concentration intracellular increase due to Na(+)/K(+)-ATPase inhibition, which leads to increased intracellular Ca(2+) concentration ([Ca(2+)](i) via a backward-running Na(+)/Ca(2+) exchanger. Contrariwise, antiproliferative effects could depend on signalling pathways induced by cardiac glycoside interaction with the Na(+) pump via intramembrane and cytosolic protein-protein interactions [53].

Signalling is initiated by interacting with neighboring membrane proteins and organized cytosolic cascades of signaling molecules. Diverse mechanisms reported as specifically involved in cardiac glycoside-mediated malignant cell-proliferation control has been compiled, are reviewed in [54-58], and include activation of ERK1/2 activation, increased cell cycle inhibitor p21Cip1 expression, and consequent cell cycle-progression inhibition (through decreased cyclin protein expression), inhibition of transcription factors such as Nuclear factor-kappaB (NF-κB) and Activator protein-1 (AP-1), inhibition of Akt and related critical phosphoinositide-3 kinase (PI3K)-pathway components, sustained Reactive oxygen species (ROS) production with consequent mitochondrial injury and reduction in expression of anti-apoptotic proteins such as Bcl-xL and Bcl-2. In addition to their antiproliferative effects, experimental evidence indicates that cardiac glycosides are effective apoptotic inducers through an increase in Fas and Tumor necrosis factor receptor 1 (TNFR1) expression and by Apo2L/TNF-related apoptosis-inducing ligand (TRAIL) in non-small-cell lung cancer [59-61]. Induction of autophagia has also been reported. Human PANC-1 pancreatic cancer cell-treated cardiac glycosides exhibit clear hallmarks of autophagy, including damaged mitochondria-associated autophagosome body formation and light chain-1 protein expression, an early indicator of autophagosome formation [62].

Interestingly, there is evidence that cardiac glycosides have a selective growth inhibitory effect on malignant over normal cells, which in part could be related with glycolysis inhibition [63,64]. Moreover, these display selective radiosensitizing properties in malignant cells [65-67]. This selectivity has yet to be studied, but may depend on alpha-subunit 1 and 3 normal and malignant tissue expression pattern. Increased expression of α3 over α1 has been observed in primary colon cancer tumors and cell lines, [68]. To the contrary, α1 subunit overexpression has been regarded as the therapeutic target in glioblastoma and lung carcinomas [69-71]. Further studies on the expression pattern of these subunits may aid in understanding the antitumor effects of digitalis and may be potentially utilized as predictive response factors.

Taken together, all this experimental evidence supports the clinical testing of cardiac glycosides despite their narrow therapeutic index. Currently, a phase II study of second-line erlotinib plus digoxin in patients with non-small-cell lung cancer is ongoing.

#### Renin-angiotensin system (RAS) antagonists as cardiovascular agents

Angiotensin II (AngII), the biologically active peptide of the renin-angiotensin system (RAS), is a major blood pressure and cardiovascular homeostasis regulator and is also recognized as a potent mitogen. AngII is an octapeptide produced by cleavage of the inactive decapeptide Angiotensin I (AngI) by Angiotensin I-converting enzyme (ACE), a zinc metalloprotease found in the circulation or bound to the cell membrane. AngI itself is produced by enzymatic cleavage of the angiotensinogen precursor by renin. In addition to plasma AngII production, a local RAS has shown to be functional in several organs, leading to production of AngII, which might have a paracrine or autocrine function. AngII mediates its biological effects through binding to two subtypes of receptors, AT_1_R and -_2_R, which belong to the G-protein-coupled receptor superfamily, but that have different tissue distribution and intracellular signaling pathways [72,73]. The majority of AngII's physiological effects have been attributed to stimulation of the AT_1_R – further subdivided into AT1aR and -2bR in rodents – whereas AT2R often functions as a counter-regulatory receptor. In addition to its effects on blood pressure, AngII has shown to play a role in various pathological situations involving tissue remodeling, such as wound healing, and cardiac hypertrophy and development [74,75].

Angiotensin-converting enzyme inhibitors (ACE-I) were introduced approximately 20 years ago as antihypertensive agents and have since become one of the most successful therapeutic approaches for high blood pressure, congestive heart failure, post-Myocardial infarction, and diabetic nephropathy. This wide range of indications is a consequence of the fact that ACE-I are thought to possess organ-protective features that extend beyond their ability to control BP. Approximately 10 years ago, the first orally active, selective antagonists of the Ang II AT_1_-receptor, the sartans, were introduced into clinical practice. These drugs differ from ACE-I in that they selectively block one of the Ang II AT receptors, the AT_1_-receptor, which is responsible for known Ang II cardiovascular actions. They do not interfere directly with kinin breakdown and leave other AT receptors, notably the AT_2_-receptor, unopposed [76,77].

These drugs are in general well tolerated. A number of agents of each class are currently clinically available. Among ACE-I, at least nine agents are commonly used, including *benazepril, captopril, enalapril, fosinopril, lisinopril, moexipril, quinapril, ramipril*, and *trandolapril*. The majority of these agents are taken orally once a day. A dry, irritating cough is the most common side effect, but angioedema is the most serious; if it affects the oropharynx, can be fatal. Angioedema is most frequently found among blacks and smokers. ACE inhibitors may increase serum K and creatinine levels, especially in patients with chronic renal failure and those taking K-sparing diuretics, K supplements, or non-steroidal anti-inflammatory drugs. ACE inhibitors are the least likely of the antihypertensives to cause erectile dysfunction, and are contraindicated during pregnancy. In patients with a renal disorder, serum creatinine and K levels are monitored at least q 3 months. Patients who have stage 3 nephropathy (estimated GFR of < 60 mL/min to > 30 mL/min) and are administered ACE inhibitors can usually tolerate up to a 30–35% increase in serum creatinine above baseline. ACE inhibitors can cause acute renal failure in patients who are hypovolemic or who have severe heart failure, severe bilateral renal artery stenosis, or severe stenosis in the artery to a solitary kidney. Similarly, there are a number of orally available angiotensin II receptor blockers such as *candesartan, eprosartan, irbesartan, losartan, olmesartan, telmisartan*, and *valsartan*. These agents may be safely begun in persons < 60 years of age with initial serum creatinine of ≤ 3 mg/dL. Adverse-event incidence is low; angioedema occurs, but much less frequently than with ACE inhibitors. Precautions for angiotensin II receptor blocker use in patients with renovascular hypertension, hypovolemia, and severe heart failure are the same as those for ACE inhibitors. Angiotensin II receptor blockers are contraindicated during pregnancy [78-83].

#### Renin-angiotensin system (RAS) antagonists as anticancer agents

Angiotensin II (AngII), the biologically active peptide of the renin-angiotensin system (RAS), is also recognized as a potent mitogen that participates in various pathological situations involving tissue remodeling. The role of AngII in cell proliferation and migration, as well as in several experimental angiogenesis models, suggests that the RAS system may be involved in tumorigenesis. Recent studies have revealed local expression of several RAS components in various cancer cells and tissues, including brain, lung, and pancreatic cancers, as well as breast, prostate, skin, and cervix carcinomas [84].

The idea that ACE inhibitors might play a protective role in cancer was suggested by observations of reduced incidence of breast and lung cancer in patients undergoing long-term treatment with the captopril, lisinopril, or enalapril [85]. Further suggestions obtained from the finding of lower cancer risk exhibited by individuals homozygous for I or A alleles at the ACE gene, which is associated with lower ACE levels [86,87], as well as lower risk of tumor progression in patients with gastric cancer carrying the polymorphism [88]. In experimental systems, the antitumor effects of diverse ACE inhibitors show that these inhibit cell proliferation and possess antiangiogenic, antimetastatic and anti-inflammatory effects [89-93]. These antitumor properties are also demonstrated by a number of sartans, selective Ang II AT_1_-receptor antagonists [94-101], further reinforcing that blockade of AT_1_R could be an effective anticancer strategy, not only because these drugs target cancer cells, but also endothelial cells at the tumor and stroma.

Major intracellular pathways that might be involved in potential AT_1_R effects in cancer cell proliferation, angiogenesis and inflammation are those whose participation in cancer is well known. AT_1_R is able to transactivate EGFR in cancer cell lines, which leads to ERK, STAT3, and PKC activation [102-105]. The known AT_1_R proangiogenic effect mainly results from VEGF, angiopoietin-2, and VEGFR2 up-regulation via EGFR transactivation [106] in tumor cells, as well as VEFG up-regulation in fibroblasts, the major stromal cellular components involved in tumor-related angiogenesis by activating NFkB, AP-1, and PKC activation. further, the AT_1_R subtype also displays anti-apoptotic effects in microvascular endothelial cells by up-regulating survivin and suppressing caspase-3 activity via phosphoinositide-3 kinase PI-3K-Akt-pathway activation [106,107]. RAS activation through AT_1_R up-regulates several inflammatory cytokines and chemokines [e.g., interleukins (IL)-6/12 and -8, and monocyte chemoattractant protein-1 (MCP-1)] via signaling pathways involving nuclear factor kappa B (NFkB), activator protein-1 (AP-1) and ROS [108,109]. Some angiotensin type 1 receptor blockers, such as telmisartan, candesartan, irbesartan, and losartan, are peroxisome proliferator-activated receptor-gamma pathway agonists; hence, this pathway may also explain some antitumor effects of these agents [110].

Thus, RAS antagonists – either ACE inhibitors or AT_1_R blockers already in use as antihypertensive drugs with mild side effects – should be considered for clinical development as anticancer treatment. To date, a pilot study in patients with hormone refractory prostate cancer has shown prostate specific antigen (PSA) changes in eight (34.8%) of 23 patients treated with candesartan 8 mg once daily. Six males with a PSA decline of > 50% demonstrated performance status improvement, and mean time to PSA progression (TTPP) in responders was 8.3 months (range, 1–24 months). Only one patient showed low blood pressure during treatment [111]. These results further support the clinical development of these classes of anticancer agents.

#### Nitroglycerin for coronary heart disease

Coronary artery disease is a leading cause of morbidity and mortality in many developed and developing countries. Management of this condition relies on risk factor modification and the use of drugs such as antiplatelets, beta blockers, nitrates, calcium channel blockers, and revascularization if symptoms persist despite medical therapy and ACE inhibitors and statins [112,113].

Nitrates improve the balance between myocardial oxygen supply and demand primarily by decreasing oxygen demand, and decreases myocardial oxygen demand by reducing preload via peripheral vein dilation. Nitrates also improve myocardial oxygen supply by dilating epicardial coronary arteries and collateral vessels, leaving resistance vessels alone [114]. Nitroglycerin (glyceryl trinitrate [GTN]), a potent smooth-muscle relaxant and vasodilator originally manufactured by Alfred Nobel, has been employed to treat angina and heart failure for > 130 years. Its main sites of action are in the peripheral vascular tree, especially in the venous or capacitance system, and in coronary blood vessels. Nitroglycerin's vasodilator effect occurs through 1,2-glyceryl dinitrate and nitrite formation by means of the mitochondrial enzyme aldehyde dehydrogenase (mtALDH), leading to cGMP production and vascular smooth-muscle relaxation [115]. Even severely atherosclerotic vessels may dilate in areas without atheroma, lowering systolic blood pressure and dilating systemic veins, thus reducing myocardial wall tension, a major determinant of myocardial O_2 _need. Sublingual nitroglycerin is administered for an acute attack or for prevention before exertion. Dramatic relief usually takes place within 1.5–3 minutes, is complete at 5 minutes, and lasts up to 30 minutes. The dose may be repeated q 4–5 minutes up to three times if relief is incomplete.

Long-acting nitrates (oral or transdermal [t.d.]) are used if symptoms persist after the β-blocker dose is maximized. If angina occurs at predictable times, a nitrate is administered to cover these times [116]. Nitroglycerin patches slowly release the drug for a prolonged effect; exercise capacity improves 4 hours after patch application and wanes in 18–24 hours. Nitrate tolerance may occur, especially when plasma concentrations are maintained constant. The most frequent side effects of nitroglycerin patches are low blood pressure (4%), postural low blood pressure, crescendo angina (2%), tachycardia, flushing, peripheral edema, headache, lightheadedness, syncope (4%), dizziness, nausea, vomiting, blurred vision, and diaphoresis [117].

#### Nitroglycerin as anticancer agent

It is well known that hypoxia confers resistance to common cancer therapies; however, it has also has been shown to result in genetic changes which may allow a survival advantage and increase the tumorigenic properties of cancer cells. Additionally, it may exert a selection pressure, allowing tumor cell expansion with a more aggressive phenotype. This adaptation is most likely a multifactorial process involving coordination of various stress-induced signaling pathways, including those regulated by hypoxia-inducible factor-1 and nuclear factor kappaB together with their resistance mechanism-linked downstream targets [118].

Experimental data suggest that treatment of several human cancer cells with nitric oxide and NO mimetic agents can effectively restore the sensitivity of resistant populations to the cytotoxic effects of chemotherapeutics both *in vitro *and *in vivo *[119-122]. To date, the specific mechanisms through which NO restores sensitivity to anticancer agents are not clearly understood. Potential mechanisms contributing to NO chemosensitizing activity include vascular changes that promote increased blood delivery and tumor oxygenation, antioxidant effects, and glutathione detoxification/redox buffering-system down-regulation, inhibition of key transcription factors such as HIF-1 and NF-kappaB, as well as drug efflux-transporter and DNA repair-enzyme inhibition [123].

NO exerts the majority of its physiological effects by binding to its guanylyl cyclase-coupled receptors in a specialized heme group, the occupation of which results in conformational changes that trigger GC activity. Thus, generation of cyclic GMP from GTP then engages various downstream targets including protein kinases, phosphodiesterases, and ion channels, giving rise to modifications in cell functions such as smooth muscle relaxation, platelet disaggregation, and synaptic plasticity. NO also regulates a wide range of biological functions via post-translational protein modification [124]. Therefore, NO's biological activities can be divided into cGMP-dependent and cGMP-independent pathways. cGMP formation is considered the main physiological signaling NO pathway [125].

The three principal cGMP targets are protein kinase G, cyclic-nucleotide-gated channels, and cyclic nucleotide phosphodiesterase [126,127]. A recent study showed that the cancer cell chemosensitivity-mediating via is the NO signaling pathway involving cGMP production and subsequent PKG activation, and that suppression of endogenous NO production (hyponitroxia) appears to be a key component of the underlying mechanism of hypoxia-induced drug resistance in cancer cells [128]. This concept is supported by several lines of evidence, such as that L-arginine conversion into L-citrulline, that NO requires molecular oxygen [129], and that exposure to low O_2 _levels (1–3%) inhibits NO production by up to 90% in endothelial cells and macrophages [130,131]. Furthermore, cGMP production is markedly decreased in hypoxia (0.5% O_2_)-incubated tumor cells for 24 hours [132]. Hypoxia has also been shown to increase arginase activity in macrophages [133], thus diverting L-arginine metabolism away from the NO generation pathway and into the urea cycle.

This experimental evidence was the basis for a double-blind phase II randomized study in which 120 patients with stage IIIB/IV NSCLC were randomly assigned to vinorelbine 25 mg/m^2 ^on days 1 and 8 and cisplatin 80 mg/m^2 ^on day 1, with t.d.-applied nitroglycerin (25 mg/patient daily for 5 days; arm A) or with placebo patch (arm B) every 3 weeks for a maximum of four cycles. Trial results indicate that nitroglycerin was able to increase the response rate significantly (72 vs. 42%), which was reflected in longer median-time-to-progression (327 vs. 185 days). It is noteworthy that there no severe side effects except for grade 1- and -2 headaches in patients treated with nitroglycerin arm [134]. Currently, a phase III trial is ongoing to confirm these results.

#### Alpha_1_-adrenoceptor antagonists as antihypertensives

Alpha_1 _adrenergic blocking drugs are effective in reducing blood pressure and accomplish this in a fashion comparable to the majority of other antihypertensive drug classes. These agents reduce blood pressure incrementally when combined with other antihypertensives and are the sole antihypertensives that improve plasma lipid profile, decrease blood viscosity and increase red-blood-cell deformability and endothelial function as well [135,136]. Prazosin was marketed in 1976 followed by doxazosin and terazosin, which are once-daily dosed and more recently, administered in a sustained release preparation. Two additional antihypertensives, tamsulosin and alfuzosin, are relatively uro-selective agents and are commonly administered to patients with benign prostatic hypertrophy [137]. Doxazosin also inhibits human vascular smooth-muscle cell proliferation and migration, independent of α_1_-adrenoceptor blockade [138]. Alpha_1_-adrenergic-specific antagonists inhibit norepinephrine's vasoconstrictor effect. They do so by selectively inhibiting post-synaptic α_1 _receptor activation by circulating and/or neurally released catecholamines, but do not inhibit presynaptic α_2_-adrenergic receptors; therefore, inhibition of additional norepinephrine release by an α_2_-adrenergic receptor stimulation feedback mechanism is preserved. Alpha_1_-adrenergic-specific antagonists do not interfere with the renin-angiotensin-aldosterone system [139]. The most troublesome side effect with α_1_-adrenergic antagonists has been first-dose low blood pressure or syncope, most frequently observed with shorter-acting agents, in volume-depleted states, and with higher doses of these compounds. Other side effects are uncommon; these drugs may produce urinary incontinence in women, but this is reversible on withdrawal of the drug. In general, α_1_-adrenergic antagonists should be used cautiously in children or during pregnancy [140,141].

Currently, α_1_-adrenergic antagonists are no longer considered suitable initial drugs for uncomplicated early-stage high blood pressure according to several guideline-generating groups, due to Antihypertensive and Lipid-Lowering Treatment to Prevent Heart Attack Trial (ALLHAT) findings. In this trial, the doxazosin-treatment arm of the study was terminated early, because increased cardiovascular endpoints were observed when compared with chlorthalidone. There was a 19% excess stroke incidence with doxazosin and a highly significant increase (25%) in combined cardiovascular disease [142,143].

#### alpha_1_-adrenoceptor antagonists as anticancer agents

It has long been hypothesized that epinephrine levels are acute and chronically elevated in response to acute or sustained stress and that such an increase is implicated in stress-related immunosuppression pathogenesis, which in turn may increase tumor incidence and promote metastatic growth [144,145]. However, despite that alpha and beta adrenergic receptors are expressed in malignant tumor tissues and that stimulation by catecholamines may exert a tumor growth effect [146-152], convincing evidence of their role in tumorigenesis continues to be lacking.

Both alpha and beta adrenergic receptors stimulate several cAMP-mediated pathways through receptor coupling to GTP-binding protein Gs [153,154]. Alpha adrenoceptors have been divided into α_1 _and -_2 _receptors. Multiple α_1 _and -_2_-adrenoceptor subtypes exist. Relevant to this discussion, three α_1 _adrenoceptor subtypes have been cloned and are designated α_1a_, -_1b_, and α-_1d_. Alpha_1 _adrenoceptors are localized postsynaptically in nerve terminal-adjacent smooth muscle. After extensive characterization of cloned and native receptors in diverse tissues, it remains difficult to ascribe a definite clinical purpose to each α_1 _adrenoceptor subtype beyond the role of α_1 _adrenoceptor stimulation in the BPH symptom profile [139].

The realization that alpha_1 _adrenergic antagonists may play a role in cancer therapy arose from observations that doxazosin and terazosin induce prostate cancer apoptosis. It has been demonstrated that treatment of prostate cancer cells with doxazosin or terazosin results in significant cell-viability loss via apoptosis induction in a dose-dependent manner, without affecting the cell proliferation rate. Interestingly, exposure to phenoxybenzamine, an irreversible alpha_1_-adrenoceptor inhibitor, does not abrogate the apoptotic effect of doxazosin or terazosin against human prostate cancer or smooth muscle cells, suggesting that doxazosin and terazosin apoptotic activity against prostate cells is independent of their capacity to antagonize alpha_1_-adrenoceptors. Furthermore, an in vivo efficacy trial demonstrated that doxazosin administration (at tolerated pharmacologically relevant doses) in Severe combined immunodeficient (SCID) mice bearing PC-3 prostate cancer xenografts resulted in significant tumor growth inhibition [155,156]. The proapoptotic effect of doxazosin results from Bax and Fas/CD95 up-regulation and Bcl-xL and TRAMP/Apo3 down-regulation as shown in a global expression assay, and can be blocked by specific caspase-8 inhibitors as doxazosin increases Fas-associating death domain-containing protein (FADD) recruitment and subsequent caspase-8 activation, implicating Fas-mediated apoptosis [157]. In addition, doxazosin inhibits human vascular endothelial cell adhesion, migration, and invasion in human endothelial cells [158]. In a recent study among several quinazoline-based alpha_1_-adrenoceptor antagonists, prazosin displayed antiproliferative activity superior to that of other alpha_1_-blockers including doxazosin, terazosin, tamsulosin, and phentolamine. Prazosin induces cell apoptosis through induction of DNA damage stress, leading to Cdk1 inactivation and G2 checkpoint arrest, as well as mitochondria-mediated apoptosis. In vitro antitumor effects are also observed *in vivo *with oral administration of prazosin in PC-3-derived cancer xenografts in nude mice [159].

More recently, doxazosin has been reported to inhibit proliferation and induce apoptosis in breast cancer cells *in vitro *in alpha_1_-adrenergic receptor-independent mechanisms. Intriguingly, doxazosin treatment reduced phosphorylated EGFR expression, decreased pERK1/2 levels, and decreased NF-kB, AP-1, SRE, E2F and CRE-mediated transcriptional activity. These effects cannot be blocked by EGF- and TNFa-treatment alone, but by the combination of EGF and TNFa treatments, indicating that doxazosin inhibits both EGFR and NF-kB signalling pathways to induce breast cancer cell apoptosis [160]. Taken together, the evidence challenges conventional knowledge of the mechanism of action of alpha_1_-adrenoceptor antagonists and points to a new therapeutic value for these drugs by providing a differential molecular basis for their anti-tumor efficacy. The fact that the majority of alpha_1_-adrenergic antagonists are quinazoline-based drugs such as gefinitib and that doxazosin treatment reduces phosphorylated EGFR and phosphorylated ERK levels – effects that overlap with those induced by gefitinib [160] – suggest that these inexpensive drugs could be as effective as current EGFR inhibitors and merit clinical testing.

#### Hydralazine as antihypertensive and vasodilator

Hydralazine, a potent arterial vasodilator that reduces peripheral resistance directly by relaxing the smooth muscle cell layer in arterial vessels, has long been utilized for management of hypertensive disorders and heart failure; nonetheless, its current use is limited nearly to hypertensive disorders during pregnancy. Despite numerous studies conducted with the drug, its mechanism of action has remained unknown. Notwithstanding this, it has been suggested that hydralazine may function by either modulating the effect of sympathetic nerve ending-released purine-like compounds and/or by producing an altered Ca^2+ ^balance in vascular smooth muscle cells [161-164].

Hydralazine is well absorbed through the gastrointestinal tract, but systemic bioavailability is low. Because the acetylated compound is inactive, the dose required to produce a systemic effect is higher in fast acetylators. N-acetylation of hydralazine occurs in bowel and/or liver. Hydralazine's half-life is 1 hour and systemic clearance of the drug is approximately 50 mL/kg/min. Systemic metabolism is dependent on hydroxylation followed by conjugation with glucoronic acid in liver, which is not dependent on acetylation rate; therefore, half-life does not differ to a great degree between slow and fast acetylators [165]. Hydralazine peak concentration in plasma and the drug's peak hypotensive effect occurs within 30–120 minutes of ingestion. Although its half-life in plasma is approximately 1 hour, duration of the hypotensive effect can last as long as 12 hours. Hydralazine's antihypertensive effect possesses no clear dose-response effects. The dose varies from 10 mg four times a day to 50 mg four times daily. After stabilization with multiple daily doses, a twice-daily dose regimen can be effective. Slow acetylators require a lower dose. For heart failure, recommended doses are higher (up to 800 mg daily or more); as a rule, 10–100 mg four times a day can be effective [166]. Common side effects include headache, nausea, flushing, low blood pressure, palpitation, tachycardia, dizziness, and angina pectoris. Hydralazine causes autoimmune reactions, among which the drug-induced lupus-like syndrome is the most common [161].

#### Hydralazine as anticancer agent

The first observations on DNA demethylation as a hydralazine off-target effect were performed in 1988 in the course of experiments to prove that this drug could induce self-reactivity in cloned T-cell lines and DNA hypomethylation [167], followed by reports on its ability to restore expression of tumor suppressor genes silenced by promoter hypermethylation in cancer cell lines and primary tumors [168-171]. *In silico *models have demonstrated that residues Lys162 and Arg 240 within the enzyme active site interact with hydralazine at distances between these residues and hydralazine nitrogen atoms not exceeding 4A°. These interactions are energetically stable, supporting that hydralazine may inhibit DNA methyltransferase [172].

Contrariwise, other authors have reported that hydralazine decreases DNA methyltransferase 1 and 3a expression in a similar manner to PD98059, a Mitogen-activated protein kinase kinase (MEK) inhibitor, this suggesting that hydralazine does not directly inhibit DNA methyltransferase enzymatic activity [173]. These discrepancies with regard to hydralazine's precise mechanism of action as DNA methylation inhibitor extends to other non-nucleoside DNA methylation inhibitors, which may stem from technical issues [174]; hence, this issue concerning hydralazine's mechanism of action needs to be further addressed. The pre-clinical and clinical development of this agent has been performed in combination with valproic acid [175]. Currently, hydralazine alone is being tested as demethylating in breast and colorectal cancer, and in combination with valproate is in phase III trials in cervical and ovarian carcinomas.

#### Procainamide as antiarrhythmic

Procainamide is a group 1A cardiac antiarrhythmic drug available in oral and i.v. preparations. By blocking Na+ channels, class I drugs primarily block the rapid inward sodium current, thereby slowing the action-potential rise rate. Procainamide increases the atria's effective refractory period, and to a lesser extent, the His-Purkinje system bundle and heart ventricles. Therapeutic procainamide levels may exert vagolytic effects and produce slight heart rate acceleration, while high or toxic concentrations may prolong A-V conduction time, or induce AV block, or even cause abnormal automaticity and spontaneous firing, by unknown mechanisms [176]. Procainamide is well absorbed following oral administration. The absolute bioavailability is approximately 85% in patients and healthy subjects. Plasma protein binding of procainamide is insignificant, approximately 20%. The apparent distribution volume is approximately 2 L/kg. Procainamide's elimination half-life is 3–4 hours in patients with normal renal function, but reduced renal function prolongs the half-life. Procainamide is mainly eliminated intact by the kidneys. The only metabolite of any significance comprises N-acetylprocainamide (NAPA), which is mainly excreted by the kidney. NAPA plasma concentration is lower than the PA concentration in the majority of individuals. The reverse may occur in individuals who form more of the metabolite while also having reduced kidney function. NAPA has significant antiarrhythmic activity. An average of 65% of the dose was recovered as intact drug in urine after i.v. PA administration. Active renal secretion is the major elimination pathway for procainamide and utilizes the base-secreting system responsible for secretion of metformin, cimetidine, ranitidine, triamterene, and flecainide; thus, there is a potential for drug-drug interactions at this level [177,178]. This drug is currently indicated for treatment of atrial fibrillation and is second choice for sustained ventricular arrhythmia management (in the acute MI setting). It is also effective in suppression of premature ventricular contractions and paroxysmal ventricular tachycardia rapidly following i.v. administration. Among its side effects, nausea and vomiting are common [179-181]. Like hydralazine, its long-term use is associated with drug-induced, reversible lupus erythematosus-like syndrome, which occurs at a frequency of 25–50%. Positive antinuclear antibody test is common, although symptoms disappear upon drug discontinuation. In slow acetylators, the procainamide-induced lupus syndrome takes place more frequently and earlier in therapy than in rapid acetylators [182].

#### Procainamide as anticancer agent

Clues from discovering the DNA methylation inhibitory activity of this drug, as from hydralazine, derived from its lupus-like properties in experimental lupus systems [183]. Afterward, in 2001 Lin et al. reported that procainamide was able to demethylate and restore GSTP1 gene expression in LNCaP prostatic carcinoma cell line *in vitro *and in nude mice carrying prostatic carcinoma xenografts [184]. These effects of procainamide were also confirmed in additional genes and cell lines as reported for hydralazine [168]. Subsequently, it was reported that procainamide specifically inhibits hemimethylase activity of DNA methyltransferase 1 (DNMT1), the mammalian enzyme thought responsible for maintaining DNA methylation patterns during replication. At micromolar concentrations, procainamide was found as a partial competitive DNMT1 inhibitor, reducing the enzyme's affinity for its two substrates: hemimethylated DNA, and S-adenosyl-l-methionine. By doing this, procainamide significantly decreased DNMT1 processivity on hemimethylated DNA. Procainamide was not a potent inhibitor of *de novo *methyltransferases DNMT3a and -b. As further evidence of procainamide's specificity for DNMT1, procainamide failed to lower genomic 5-methyl-2'-deoxycytidine levels in HCT116 colorectal cancer cells when DNMT1 was genetically deleted, but significantly reduced genomic 5-methyl-2'-deoxycytidine content in parental HCT116 cells and in HCT116 cells in which DNMT3b was genetically deleted [185]. No clinical studies of procainamide as demethylating agent are reported.

### Local anesthetics

#### Procaine as local anesthesic

Procaine is a local anesthetic drug of the amino ester group that was introduced in 1905 and became the first local anesthetic to gain wide acceptance in the U.S. Nonetheless, its popularity as a local anesthetic declined after the introduction of lidocaine in 1948, which is the most frequently used local anesthetic at present. Procaine is currently used primarily to reduce the pain of intramuscular injection of penicillin and is also used in dentistry. Local anesthetics block nerve-impulse generation and conduction, presumably by increasing the nerve's electrical excitation threshold by slowing propagation of the nerve impulse and by reducing the action-potential rise rate [186]. Systemic absorption of local anesthetics produces effects on the cardiovascular and central nervous systems. At blood concentrations achieved with normal therapeutic doses, changes in cardiac conduction and peripheral vascular resistance are minimal. Nevertheless, toxic blood concentrations depress cardiac conduction and excitability, which may lead to atrioventricular block and ultimately, to cardiac arrest. In addition, myocardial contractility is depressed and peripheral vasodilation occurs, leading to decreased cardiac output and arterial blood pressure. At the central nervous system, local anesthetics can produce stimulation, depression, or both, manifested by restlessness, tremors and shivering, convulsions, followed by depression, and coma progressing ultimately to respiratory arrest [187,188]. Depending on the administration route, local anesthetics are distributed to some extent to all body tissues and bind plasma proteins at varying degrees. Several pharmacokinetic parameters of local anesthetics can be significantly altered by the presence of hepatic or renal disease, addition of epinephrine, factors affecting urinary pH, renal blood flow, the drug administration route, and age of the patient. [189].

#### Procaine as anticancer agent

Procaine, like procainamide, is a derivative of 4-aminobenzoic acid, but the former is the ester with 2-(diethylamino) ethanol, while the latter is the amide with 2-(diethylamino) ethylamine. Its demethylating activity, therefore, was suggested by its structural analogy to procainamide, and was demonstrated in 2003 by Villar-Garea et al., who reported that procaine leads to global genomic DNA hypomethylation and demethylation and reactivation of tumor suppressor genes with hypermethylated CpG islands in MCF-7 breast cancer cells. These effects of procaine are associated with growth inhibitory effects in these breast cancer cells. Although that procaine inhibits DNA methyltransferase activity was not demonstrated, it probably does, because procaine, like procainamide, binds strongly to CpG-rich DNA [190]. Procaine has also shown to inhibit growth and to reactivate the expression of RASSF1A mRNA in nasopharyngeal cancer cell lines [191], as well as to reactivate estrogen receptor-gene expression in MCF-7 breast cancer cells [192]. No clinical studies of procaine as demethylating agent are reported; instead, analogs of this drug are pursued to exploit its demethylating activities [193].

### Antiepileptics

#### Valproic acid as antiepileptic

Valproic acid (VPA) is a small, branched fatty acid whose chemical properties allow easy delivery to the organism and cells. It is slightly soluble in water, highly soluble in organic solvents, and stable at room temperature. Because valproic acid exists in a dissociated form in alkali metal-containing water solutions, it can be easily delivered to organisms in the form of sodium or magnesium salts, which are water soluble. Yet the two preparations are bioequivalents; magnesium valproate appears to be a drug without bioavailability problems and with reduced intersubject variability, compared with that of sodium valproate [194]. Valproic acid is now an established drug for treatment of epileptic seizures and mania in bipolar disorder. In the human brain, valproic acid affects neurotransmitter GABA function by potentiating GABA inhibitory activity by several means, including inhibition of GABA degradation, increased GABA synthesis, and decreased GABA turnover. It was also found to attenuate NMDA-mediated excitation, block voltage-dependent Na+ channels, and modulate neuron firing frequency [195,196].

VPA is rapidly absorbed after oral administration, with peak serum levels occurring approximately 1–4 hours after a single oral dose. Valproic acid half-life in serum falls typically within the range of 7–16 hours. When the drug is administered with meals, a slight delay in absorption occurs, but this does not affect total absorption. VA distribution throughout the body is rapid. The drug is strongly bound (95%) to human plasma proteins. Decreases in the extent of protein binding and variable changes in valproic acid clearance and elimination may result from dosage increases. As an antiepileptic, the therapeutic plasma concentration is believed to range from 50–100 μg/mL. VPA is primarily metabolized to the glucoronide conjugate in the liver. Only very little unmetabolized parent drug is excreted in urine. VPA and its metabolites are eliminated mainly in urine, with minor amounts appearing in feces. VPA is in general well tolerated by patients. Neurological side effects such as sedation, dizziness, and tremor, as well as mild gastrointestinal toxicities, usually take place early during treatment [195-197]. The most serious adverse events are liver failure and teratogenicity. Fatal hepatotoxicity is rare (approximately 1:15,000) and principally occurs in children aged < 2 years treated with multiple drugs. It can induce birth defects such as neural tube closure defects and other malformations when administered during early pregnancy. Teratogenicity and antiepileptic activity appear to require different mechanisms of action, because molecule modifications generate selective compounds with either teratogenic or antiepileptic activity [198,199].

#### Valproic acid as anticancer agent

The finding that VPA was an effective inhibitor of HDACs arose from observations that valproic acid was able to relieve transcriptional repression of a peroxisomal proliferation and activation of a glucocorticoid receptor (GR)-PPARδ hybrid receptor and a RAR-dependent reporter gene expression system, suggesting that VPA acts on a common factor in gene regulation, such as co-repressor-associated HDACs, rather than on individual transcription factors or receptors. Consistent with this finding, it was shown that VPA causes N-terminal tail hyperacetylation of histones H3 and -4 *in vitro *and *in vivo *and was proven to inhibit HDAC enzymatic activity directly at a VPA concentration of 0.5 mM [200].

Simultaneously, Phiel et al., after demonstrating VPA's ability to activate multiple promoter-regulated transcription, assayed HDAC1 activity in the presence of VPA in HeLa cells that over-expressed HDAC1. As expected, VPA inhibits HDAC1 *in vitro *in a dose-dependent manner, with an of 0.4 mM, falling within the therapeutic range for VPA therapy in humans. The authors also demonstrated that VPA inhibits HDACs other than HDAC1, including HDAC1, -2, -3, -4, and -8 with a 50% inhibition between 0.5 and 2 mM. VPA-induced hyperacetylation of H4 and non-histone proteins such as p53 was also demonstrated at concentrations as low as 1–2 mM [201]. Later, employing a series of compounds with structural similarity to VPA, Gurvich et al. found that VPA inhibits class I HDACs (HDACs 1–3) with IC_50 _values ranging from 0.7–1 mM and inhibits class II subclass I HDACs 4, -5, and -7 with IC_50 _values ranging from 1–1.5 mM; to the contrary, VPA does not inhibit HDAC 6 or -10 (class II subclass II). Interestingly, relative VPA-analog potencies to inhibit HDACs correlated with their potencies in inducing leukemia cell-line differentiation, which led the authors to conclude that VPA effects on differentiation are most likely due to inhibition of HDACs [202]. Further, it has additionally been shown that VPA alters the expression of genes that regulate chromatin structure. VPA in breast cancer cells induces a depletion of several members of structural maintenance of chromatin (SMC) proteins, SMC-associated proteins, DNA methyltransferase, and heterochromatin proteins, which lead to chromatin decondensation, enhanced DNA sensitivity to nucleases, and increased DNA interaction with intercalating agents. This modulation is not a direct – but is rather a downstream – effect of histone acetylation reversible upon drug withdrawal [203].

VPA has shown potent antitumor effects in a variety of *in vitro *and *in vivo *systems by modulating multiple pathways including cell cycle arrest, apoptosis, angiogenesis, metastasis, differentiation, and senescence. These effects appear to be cell type-specific, which may also depend on the differentiation level and the underlying genetic alterations [204,205]. In addition, whole genome expression by microarray analysis from primary tumors of VPA-treated patients demonstrate significant up-regulation of hundreds of genes belonging to multiple pathways including ribosomal proteins, oxidative phosphorylation, MAPK signaling, focal adhesion, cell cycle, antigen processing and presentation, proteasome, apoptosis, PI3K, Wnt signaling, calcium signaling, TGF-beta signaling, and ubiquitin-mediated proteolysis, among others [175]. Currently, there are several clinical phase I and II studies reported that show that valproic acid induces histone hyperacetylation and HDAC activity inhibition in both tumors and the peripheral blood of patients, and when associated with chemotherapy exhibits encouraging antitumor responses in a range of solid tumors and malignant hematological diseases. In addition, a number of clinical trials are ongoing with VPA in combination with DNA methylation inhibitors, classical cytotoxics, newer targeted agents, and radiation [175,206].

#### Topiramate, levetiracetam, and carbamazepine

The finding that VPA is an HDAC inhibitor led researchers to assess comparatively the activity of traditional and newer AEDs such as HDAC inhibitors in HeLa cells by immunoblot utilizing an acetylated histone H4-specific antibody, and by direct HDAC inhibition employing HeLa nuclear extract as an HDAC source and an acetylated lysine substrate. Results disclosed that the HDAC inhibitory capacity of AED is not limited to VPA, but is also shown by topiramate and a levetiracetam metabolite [207]. Although in this system carbamazepine failed to show HDAC inhibition, in a subsequent report this drug induced histone H4 acetylation in the HepG2 liver carcinoma cell line and inhibited HDAC 3 and -7, which are representative of HDAC class I and II, respectively. Quantitative testing in an *in vitro *assay demonstrated HDAC inhibition with an IC50 of 2 μM. The major active metabolite of CBZ, CBZ-10,11-epoxide, was found to have the same HDAC inhibitory activity. It is noteworthy that the IC50 of 2 μM is considerably lower than therapeutic plasma levels typically achieved in patients (range, 25–51 microM) [208]. The potential antitumor properties of these HDAC inhibition-derived AED have not been further pursued but this novel off-target effect should not be neglected in cancer research.

### Antidiabetic agents

#### Thiazolidinediones (TZDs) as antidiabetic drugs

TZDs are a relatively new class of oral agents that have rapidly gained wide usage, with an estimated 20 million prescriptions written in 2004. These compounds are effective, generally well tolerated, and complement other antidiabetic drugs [209]. The first of this drug class, troglitazone, was introduced in 1997, but was removed from the market due to the occurrence of idiosyncratic liver injury [210]. Currently rosiglitazone and pioglitazone are used as antidiabetic agents. This novel class of drugs increases insulin-stimulated glucose uptake by skeletal muscle cells; therefore, these agents decrease insulin resistance in peripheral tissues. Contrary to other antidiabetic drugs, TZDs do not stimulate insulin secretion by pancreatic islet cells [211,212]. In addition to their ability to lower insulin levels, TZDs possess certain lipid benefits. High-density-lipoprotein (HDL) cholesterol concentrations, for instance, increase with TZD therapy, and triglyceride concentrations frequently fall [213,214].

Rosiglitazone and pioglitazone appear equally effective in achieving glycemia reductions. In controlled trials, TZDs generally lower HbA1c to the same degree as other antidiabetic agents. Head-to-head comparisons have been performed on TZDs vs. metformin and sulfonylureas, with similar reductions in HbA1c [215-217]. TZDs also have a number of anti-atherogenic effects independent of their influences on glucose and insulin metabolism. These improve lipid profiles, lower blood pressure, possess antiinflammatory properties, improve endothelial function, and increase large artery compliance in patients with type 2 diabetes mellitus (DM2) [218,219].

Adverse effects of TZDs include weight gain, which appears to involve mainly peripheral subcutaneous sites, with a reduction in visceral fat depots, the latter better correlated with insulin resistance. Edema can also occur. Both weight gain and edema are more common in patients who receive TZDs with insulin. Anemia may also occur infrequently. TZDs, unlike troglitazone, have not been convincingly associated with liver injury. Patients with advanced congestive heart failure and those with hepatic impairment should not receive TZDs. Recent studies in rodent models indicate that exposure to TZD reduces bone mass compared with controls [220-222].

#### Thiazolidinediones as anticancer agents

Peroxisome proliferation activated receptors (PPARs) are members of the nuclear hormone receptor superfamily of ligand-activated transcription factors related with retinoid, steroid, and thyroid hormone receptors [223]. The PPAR subfamily consists of three members: PPAR-α; -δ (also known as β), and -γ, which have a similar structural organization. An N-terminal region responsible for ligand-independent activation followed by a DNA-binding domain of two zinc fingers separated by a linker region and the C-terminal ligand-binding domain [224,225]. The name PPARs derives from the observation that activation by xenobiotics of the first receptors to be characterized, PPAR-α, results in peroxisome proliferation in rodent hepatocytes.

PPAR-δ or -γ isoform activation, however, does not elicit this response. The majority of tissues in humans express all three receptor subtypes, although there is considerable variability in relative expression levels. PPAR-δ is expressed ubiquitously in all adult mammal tissues, whereas two PPAR-γ isoforms, γ_1 _and -_2_, which arise from alternative promoter usage and differential splicing, are preferentially expressed in adipose tissue. PPARs regulate gene expression by binding as heterodimers with retinoid × receptors (RXRs) to specific response elements (PPREs) in target-gene promotor regions. In the absence of PPAR gamma ligands, which include long-chain polyunsaturated fatty acids, eicosanoid derivates, and oxidized lipids, high-affinity complexes are formed between the PPAR-RXR heterodimer and nuclear receptor co-repressor proteins. These prevent transcriptional activation by sequestration of the nuclear receptor heterodimer from the promoter. The co-repressors dissociate as a consequence of ligand binding-induced conformational change, and the activated heterodimer can bind to PPREs. Both the soluble and DNA-bound PPAR-RXR heterodimer then recruit co-activator proteins in a ligand-dependent fashion that couples the receptor complex to other transcriptional-machinery components [226-228].

The anti-proliferative, proapoptotic, and differentiation effects of PPAR-γ activators suggest that these compounds might be useful as anticancer therapy. In support of this hypothesis, TZDs exhibit *in vitro *and *in vivo *antitumor effects on a number of malignancies including breast, lung, glioblastoma, thyroid, and urological- and gastrointestinal organ-derived malignancies [229-232]. Furthermore, PPAR-γ ligands have been shown to be potent angiogenesis and metastasis inhibitors [233-236]. Nevertheless, it remains poorly understood how PPARs γ affects malignant tumors, because they exert pleiotropic effects on cell fate and metabolism and may act both as neoplasia promoter and suppressor. In fact, and paradoxically, recent studies have shown that PPAR-γ inhibition by PPAR-γ-specific antagonists also induce cell death, apoptosis, and anoikis and inhibit tumor cell invasion [237-239].

PPAR-γ activation antitumor effects by ligands appear to be mediated by means of both PPARγ-dependent and -independent (off-target) pathways, depending on agonist type, concentration, and tumor cell type [240]. In addition, it appears that TZDs possess inhibitory effects not only on tumors cells, but also on stromal cells, such as smooth muscle, endothelial, and inflammatory cells, which explain observations on minimal-to-no inhibitory activity on some tumor cell lines *in vitro *but potent inhibition of these tumors *in vivo *[235]. Antitumor effects by a PPARγ-independent (off-target) pathway and/or a biphasic effect have also been suggested [241]. While the PPARγ agonist 15d-PGJ2 increases transcriptional activity and CD36, the antagonist GW9662 reduces this but does not block agonist-induced apoptosis in breast cancer cells. In addition, the GW9662 antagonist enhances the agonist rosiglitazone's inhibitory effect on breast cancer cells rather than rescuing tumor growth, suggesting that PPARγ activation may not be involved in TZD-caused survival and cell growth inhibition. Similar results were obtained in studies in which PC3, CaCO-2, and T47D cancer cells were inhibited by both PPARγ agonists and antagonists separately and in co-treatments [242,243]. This apparent paradoxical synergism between agonists and antagonists is in line with the finding that while PPARγ agonists can possess tumor suppressing effects, antagonists also can induce apoptosis in cancer cells [237-239].

These experimental findings have been translated into early clinical trials beginning with a successful clinical report on three cases of patients with intermediate-to-high-grade liposarcoma in whom troglitazone administration induced histologic and biochemical differentiation *in vivo *as shown by extensive tumor-cell lipid accumulation and substantial NMR-detectable tumor triglyceride increases compared with pretreatment biopsies, accompanied by marked reduction in Ki-67 expression [244].

Subsequently, however, clinical trials in patients with liposarcoma, advanced breast, and colorectal tumors demonstrated no clinical response using troglitazone or rosiglitazone [245-247]. Nonetheless, additional clinical data demonstrate that short-term rosiglitazone therapy in patients with early-stage breast cancer leads to local and systemic effects on PPARγ signaling [248], as well as radioiodine uptake in some patients with thyroglobulin-positive and radioiodine-negative differentiated thyroid cancer [249].

#### Metformin as antidiabetic drug

Metformin is an oral antihyperglycemic agent that has been widely used in non-insulin-dependent diabetes mellitus management. Considered an insulin sensitizer because it lowers glucose levels without increasing insulin secretion, metformin is distinguished from thiazolidinediones by its primary site of action. Metformin lowers endogenous glucose production at the level of the liver, while thiazolidinediones work primarily in peripheral tissues such as muscle and fat [250]. Thus, metformin's glucose lowering effect results from a 25–30% endogenous glucose-rate decrease, which is entirely accounted for by reduction in the rate of gluconeogenesis [251].

Decreased hepatic gluconeogenesis can occur by hepatic lactate uptake inhibition [252], although other studies have found that metformin decreases gluconeogenic flux through pyruvate carboxylase inhibition, phosphoenolpyruvate carboxykinase activity, and, possibly through increased conversion of pyruvate to alanine [253]. In addition, metformin also stimulates AMP-activated protein kinase (AMPK) in intact cells and in vivo, and possibly inhibits complex 1 of the mitochondrial respiratory chain. AMPK is the downstream component of a protein kinase cascade that acts as a cellular energy sensor. Once activated by ATP depletion, this turns ON ATP-producing catabolic pathways and switches OFF ATP-consuming anabolic pathways, both directly via metabolic enzyme phosphorylation and indirectly via gene expression effects. Metformin stimulates phosphorylation of a key regulatory site in the AMPK catabolic subunit in intact cells [254,255].

The oral bioavailability of this drug ranges between 40 and 60%; it is primarily excreted unchanged in urine with neligible metabolism [256]; approximately 20–30% of the drug is recovered unchanged in feces [257]. It is mainly absorbed in the upper part of the intestine, and estimated time for its complete absorption is approximately 6 hours [256]. Clinical trials with metformin have demonstrated decreased bioavailability at higher doses, suggesting saturable intestinal absorption [256,258]. Metformin is the first-line pharmacotherapy in the treatment of overweight or obese patients with DM2, with beneficial effects on weight in this population in that metformin exerts a positive effect on metabolic parameters such as waist circumference, fasting insulin, glucose levels, and triglycerides [259]. Excess-weight disorders are characterized by an increased mass of adipose tissue. The foremost physical consequences comprise impaired glucose tolerance, white-coat hypertension or high blood pressure, dyslipidemia, and coronary heart disease [260]. Some authors have suggested a benefit role of metformin in the treatment of excess weight and associated disorders [261,262]. Metformin has also been utilized as treatment in Polycystic ovary syndrome (POS), achieving promising results in terms of normalization of LH/FSH ratio, fasting insulin, testosterone, and progesterone levels, and/or pregnancy [263]. Metformin is usually well tolerated and the most frequent side effects comprise gastrointestinal effects including nausea, diarrhea, abdominal pain, and anorexia. Metformin may also cause lactic acidosis, particularly in patients with renal or liver dysfunction.

#### Metformin as anticancer drug

The discovery of LKB1 as the tumor suppressor gene responsible for Peutz-Jegher syndrome, an autosomal-dominant disorder characterized by melanocytic macules of the lips, multiple gastrointestinal hamartomatous polyps, and an increased risk for various neoplasms including gastrointestinal cancer led to the suspicion that metformin may exhibit antitumor properties, because LKB1 is an upstream AMPK regulator [264]. These observations were supported by two reports linking treatment with metformin in patients with diabetes with a lower risk of cancer [265,266].

A number of experimental data indicate that metformin AMPK exerts its antitumor actions by activating AMPK. This serine/threonine kinase consists of a heterotrimeric complex comprising a catalytic α subunit and regulatory β and γ subunits [267]. AMPK is activated under conditions that deplete cellular ATP and elevate AMP levels such as glucose deprivation, hypoxia, ischemia, and heat shock, which are associated with an increased AMP/ATP ratio [268]. AMPK actions appear to be mediated by means of multiple mechanisms. AMPK activation leads to cell cycle arrest via p53-p21 axis up-regulation, although Cyclin D1 down-regulation may also occur independently of AMPK activation [269] and protein synthesis-regulation inhibition of the TSC2-mTOR (mammalian target of rapamycin) pathway. In addition, AMPK activation impedes de novo fatty acid synthesis, specifically the generation of mevalonate, as well as other products downstream of mevalonate in the cholesterol synthesis pathway. Thus, the AMPK signalling network contains a number of tumor suppressor genes including LKB1, p53, TSC,1 and -2, and overcomes growth factor signalling from a variety of stimuli (via growth factors and by abnormal regulation of cellular proto-oncogenes including PI3K, Akt, and ERK [270].

Recent studies have reported that extracellular hormonal stimulation by adiponectin and leptin, both of which are adipose tissue-secreted peptide hormones, also could activate AMPK [271]. Adiponectin has been reported to inhibit vascular SMC proliferation [272]. Plasma adiponectin has been shown as decreased in patients with carcinomas from breast, endometrium, and stomach [273-275]. Interestingly, potential anticancer effects of adiponectin have been demonstrated in breast and endometrial cancer cells [276,277].

Thus, metformin exhibits pleiotropic effects on cancer cells as reflected by its antitumor effects in a wide variety of cancer cell lines *in vitro *and *in vivo *including breast, glioma colon, ovarian, and prostate [278-281]. Whether their antitumor actions depend on AMPK activation or whether these are independent of this pathway requires further study. What is clear, however, is that metformin possesses full potential as a cancer drug that should be fully evaluated in pre-clinical and clinical studies.

### Obesity drugs

#### Orlistat for obesity

Obesity and overweight are highly and increasingly prevalent chronic conditions. In addition to lifestyle modification as initial treatment, orlistat, a gastrointestinal lipase inhibitor, sibutramine, a centrally acting monoamine reuptake inhibitor, and rimonabant, an endocannabinoid receptor antagonist, are approved for long-term treatment of obesity [282].

Orlistat is a potent inhibitor of Fatty acid synthase (FAS) activity, a key metabolic enzyme responsible for the terminal catalytic step in *novo *fatty acid biosynthesis [283]. Orlistat was approved by the FDA as an antiobesity drug. It is a semi-synthetic derivative that irreversibly inhibits pancreatic and gastric lipases within the gastrointestinal tract [284]. Unlike other medicaments previously approved for obesity treatment, orlistat does not act on the CNS; instead, it decreases dietary fat absorption in the gastrointestinal tract by approximately 30% [285]. In addition to its antiobesity effects, orlistat reduces the incidence of DM2 mainly in patients with impaired glucose tolerance at baseline. Compared with placebo, orlistat also significantly reduces waist circumference, Body mass index, Systolic blood pressure, diastolic blood pressure, fasting glucose, and hemoglobin A_1C _concentrations in patients with diabetes, and total cholesterol, Low-density-lipoprotein cholesterol (LDL-C), and High-density-lipoprotein cholesterol (HDL-C) concentrations [286].

Orlistat possesses extremely low oral bioavailability, and when co-administered with other agents, has demonstrated no pharmacokinetic and pharmacodynamic interactions with drugs such as glyburide, digoxin, warfarin, oral contraceptives, nifedipine, and ethanol. However, orlistat interferes with the absorption of many drugs (such as warfarin, amiodarone, cyclosporine, and thyroxine, as well as fat-soluble vitamins), affecting their bioavailability and effectiveness [287].

Gastrointestinal events such as oily stools, diarrhea, abdominal pain, and fecal spotting are common. A few cases of serious adverse hepatic effects (cholelithiasis, cholestatic hepatitis, and subacute liver failure) have been reported. The majority of these events is mild to moderate in intensity, transient in duration, and decreased considerably during the second year of treatment.

Orlistat is not significantly absorbed into the systemic circulation and is well tolerated. It has been also reported that orlistat has no significant effects on calcium, phosphorus, magnesium, iron, copper, or zinc balance or on bone biomarkers [288,287].

#### Orlistat as anticancer drug

It is now clear that cancer cells possess not only high rates of aerobic glycolysis, high rate of energy-consuming processes driving increased DNA and protein synthesis, but also increased *de novo *fatty acid (FA) synthesis, a forgotten glycolytic pathway-related process [289]. There are two sources of FAs for animal metabolism: Dietary FAs, and endogenously synthesized fatty acid synthase (FASN)-catalyzed FAs utilizing acetyl-CoA as primer, malonyl-CoA as two-carbon donor, and NADPH as reducing equivalent. The predominant product of FASN is the 16-carbon FA, palmitate. Under normal conditions, dietary fat suffices to fulfill requirements with the consequent under-use of endogenous Fas [290]. Contrariwise, tumors and their precursor lesions unexpectedly undergo exacerbated endogenous FA biosynthesis irrespective of extracellular lipid levels. Tumor cells can redirect the excess glycolytic end-product pyruvate toward *de novo *FA synthesis, which is necessary to maintain a constant supply of lipids and lipid precursors to fuel membrane production and lipid-based post-translational protein modification in a highly proliferating cell population. This exacerbated lipogenesis in tumor cells is reflected by the significantly increased activity and expression of several lipogenic enzymes, of which FASN is the key terminal catalytic step in FA synthesis. Immunohistochemical studies have reported extremely high FASN levels in many pre-neoplastic lesions and cancers including breast, colorectum, prostate, bladder, ovary, esophagus, stomach, lung, oral tongue, oral cavity, head and neck, thyroid, and endometrium, and also in mesothelioma, nephroblastoma, retinoblastoma, soft tissue sarcomas, melanoma, and hepatocellular carcinoma [291,292].

FASN over-expression may actively contribute to malignant-phenotype development, maintenance, and/or promotion, because its inhibition by orlistat induces cell-cycle arrest and apoptosis in a wide variety of cancer cell lines including prostate, breast, gastrointestinal, chronic lymphocytic leukemia, and others [293-296], suggesting a role for FASN in the molecular integration of a number of signalling pathways that functionally link metabolism, proliferation, and survival in malignant cells. Second, FASN has the ability to regulate specifically the activity and/or expression of key cancer-related signalling networks of growth factors and their receptors, as well as of steroid hormones and their receptors [291]. This is of particular relevance in breast cancer cells over-expressing Her2 in which micromolar concentrations of orlistat are able to down-regulate Her2 by > 90%. In addition, orlistat in combination with trastuzumab exhibits a strong synergistic antitumor effect [297,298].

### Cholesterol-lowering agents

#### Statins as cholesterol-lowering agents

Coronary heart disease (CHD) is a major cause of morbidity and mortality worldwide. Elevated LDL-C and reduced HDL-C levels are well recognized CHD risk factors, with recent evidence supporting the benefits of intensive LDL-C reduction on CHD risk. Statins are a broadly used group of cholesterol-lowering agents that act by inhibiting the enzyme 3-hydroxy 3-methylglutaryl CoA (HMG CoA) reductase, which catalyzes the rate-limiting step in cholesterol biosynthesis [299,300]; therefore, statins reduce the concentration of downstream metabolic by-products including mevalonate, which in turn leads to increased LDL-receptor expression in hepatocytes and to increased LDL-C uptake from the circulation. Statins also tend to reduce apolipoprotein B and A-I production, as well as additional antiinflammatory effects [301-303].

Lovastatin, simvastatin, pravastatin, fluvastatin, and atorvastatin are available in most parts of the world. Lovastatin, simvastatin, and pravastatin are all fungal derivatives, whereas fluvastatin and atorvastatin are synthetic. Lovastatin and simvastatin are prodrugs and are converted into their active forms (β-hydroxy acid) in the liver, whereas the others are active in their parent forms. Concentration-dependent HMG-CoA reductase inhibition in human pharmacodynamic studies is based principally on plasma LDL-C, which takes 4–6 weeks to show a reduction after initiation of statin treatment. Fluvastatin, lovastatin, pravastatin, and simvastatin have similar pharmacodynamic properties; all can reduce LDL-C by 20–35%. The liver is the target organ for the statins, in that it is the major site of cholesterol biosynthesis, lipoprotein production, and LDL catabolism. Adverse HMG-reductase inhibitor effects during long-term treatment may depend in part upon the degree to which they act on extrahepatic tissues. Therefore, pharmacokinetic factors such as hepatic extraction and systemic exposure to (an) active compound(s) may be clinically important when comparing statins. After absorption, each undergoes extensive hepatic first-pass metabolism. Up to five primary metabolites are formed, some of which are active inhibitors. However, statins differ in certain important aspects concerning degree of metabolism and number of active and inactive metabolites. The rather extensive metabolism by different cytochrome P450 isoforms also renders it difficult to characterize these drugs with regard to tissue selectivity unless all metabolites are well characterized. HMG-CoA reductase-inhibitor availability is limited by extensive first-pass metabolism. The CYP system is responsible for the majority of the clearance of this class of drugs, with the exception of pravastatin, in which renal clearance also plays a major role in its elimination. Therefore, CYP isozyme inhibitors may significantly raise HMG-CoA reductase-inhibitor serum levels. Lovastatin, simvastatin, and atorvastatin are primarily oxidized by CYP3A4. Fluvastatin is predominately (50–80%) inactivated by CYP2C9, but CYP3A4 and -2C8 also contribute to its biotransformation. Pravastatin is not metabolized extensively by CYP isozymes, but is selectively taken up by the sodium-independent bile acid transporter. Caution must be exercised with concurrent administration of drugs that interfere with the CYP system in the presence of HMG-CoA reductase inhibitors.

Elimination half-lives range from 0.5–3.5 hours and excretion is mainly via feces [304-306]. The common side effects associated with these drugs are relatively mild and often transient in nature. The only well documented, consistent adverse effects associated with statins are muscle toxicity, including myopathy and rhabdomyolysis, and effects on liver enzymes; however, these effects are uncommon and generally resolve rapidly when treatment is stopped [307,308].

Results from large randomi**z**ed controlled trials of statin treatment have now provided confirmation that reducing cholesterol and maintaining low cholesterol levels for at least 5 years is not only safe but beneficial in the ability of statins to reduce the risks of vascular death, non-fatal MI, stroke, and the need for arterial revascularization procedures [309]. In these trials, the extent of risk reduction was judged as directly proportional to the degree to which LDL-C was lowered, consistent with this being the main mechanism [310,311]. Cholesterol-lowering is now recommended for a wide range of persons with cardiovascular risk, including those with average and below-average lipid levels [312]. This change is leading to increased statin use and utilization of more intensive regimens.

#### Statins as anticancer agents

The mevalonate pathway is now considered an important target for anticancer therapy, because its end-products are critical for functioning in both normal and cancerous cells. These products include geranylgeranyl pyrophosphate and farnesyl pyrophosphate [313]. Geranylgeranyl- and farnesyl transferase employ geranylgeranyl pyrophosphate and farnesyl pyrophosphate, respectively, for post-translational modifications of a wide variety of cellular proteins. In this activation step, farnesyl or geranylgeranyl moieties are coupled with the protein, resulting in a farnesylated or geranylgeranylated protein. This type of protein activation is referred to as (iso)prenylation. Several proteins involved in signalling are dependent on prenylation for their activity, such as Ras, nuclear lamins, transducin c, rhodopsin kinase, Rho, and all of the remaining heterotrimeric Gs [314,315]. These proteins regulate cell proliferation, intracellular trafficking, and cell motility, and this post-translational modification functions as a membrane anchor critical for their activity [316,317]. There are several molecules being studied as anticancer therapy that interfere with the mevalonate pathway. These include farnesyl transferase inhibitors, geranylgeranyl transferase inhibitors, dual inhibitors, bisphosphonates, and statins, among others [313]. Blockade of this pathway by statins results in decreased levels of mevalonate and its downstream products, influencing many critical cellular functions.

Malignant cells appear highly dependent on sustained availability of mevalonate pathway end-products [318]. Deregulated or elevated HMG-CoA reductase activity has been shown in colorectal and gastric carcinomas, and leukemia [319-321]. There are a number of pre-clinical studies showing the antineoplastic effects of statins in *in vitro *and *in vivo *systems against a number of cell lines from solid and hematological malignancies. In general and depending on cell type and experimental conditions, statins exert growth arrest, apoptosis, antimetastatic and antiangiogenic effects [322-325].

The antitumor mechanisms of statins are not yet well defined. The most studied and perhaps the most important effect lies at the isoprenylated protein level. Farnesylated Ras proteins are associated with mitogenic signal transduction in response to growth factor stimulation [326], whereas Rho subfamily proteins such as Rho, Rac1, and Cdc42, regulate signal transduction from receptors in the membrane in a variety of cellular events related with cell morphology, cell adhesion, cell motility, cell growth, and cancer cell metastasis [327,328]. RhoA and -C are posttranslationally modified by geranylgeranylation, whereas RhoB can be farnesylated and geranylgeranylated [329,330]. Thus, the majority of authors consider inhibition of prenylation of these oncogenic proteins with their consequent loss of function, due to the mechanism that causes statin-induced effects on proliferation and apoptosis. Statins also affect the Raf/MEK/ERK pathway in a cell type-specific manner [331,332] and could also affect PI3K-AKT pathways as farnesyltransferase inhibitors do [333,334]. In addition, statins affect G1/S transition control by over-expression at either the mRNA or protein level of Cdk inhibitors such as p16, -21, and -27 [335-337]. Other authors have suggested a p21- and p27-independent pathway for the effects of lovastatin. It has been observed that proteasome inhibitors partially prevent lovastatin-induced E2F-1 degradation, suggesting that lovastatin modulates E2F-1 proteasomal degradation, which may be a critical regulatory mechanism of lovastatin-induced effects [338], and that mevastatin inhibits cdk2 activity in PC3 cells through Thr-160 phosphorylation inhibition of cdk2 [339].

The vast number of patients receiving statins for hypercholesterolemia and pre-clinical evidence of their potential antitumor effects has led to its evaluation in case-control studies and meta-analyses for cancer incidence and their effects on surrogate markers of cancer. As summarized by Hindler et al. regarding statin use and cancer risk, three studies (two with pravastatin and one with all statins) have found a discrete increase in breast cancer and overall cancer incidence. However, five studies found no changes in cancer risk, whereas in five studies a large decrease in melanoma, colorectal, breast, uterine, and prostate cancer, respectively, was found [332]. Further support for statin anticancer activity of statins derives from a small prospective study to assess the effect of statin treatment on serum prostatic specific antigen (PSA) in a cohort of airline pilots from 1992–2001. Despite that serum PSA was significantly higher in the treatment group (*p *= 0.05), there was no significant difference between the groups on subsequent follow-up, while a 41.6% decrease of PSA in the treated group was observed [340]. It was also found that statin use was associated with reduced Breslow thickness or delayed metastasis of melanoma in a case-control study including 1,318 cases and 6,786 controls from The Netherlands [341].

Several phase I studies have been conducted. Tolerability of pravastatin added to idarubicin and high-dose Ara-C has been proven. In fact, the combination's maximum tolerated dose was not reached in the study, despite the use of very high doses of pravastatin (up to 1,280 mg per day). Response rates were encouraging, with a high number of patients obtaining complete responses [342]. Likewise, simvastatin administered for 7 days prior to chemotherapy, VAD, or CHOP for myeloma and non-Hodgkin lymphoma, respectively, is safe up to a dose of 15 mg/kg [343]. Lovastatin was administered in a dose-escalating trial in subjects with advanced malignancies. Lovastatin was administered at doses ranging from 10–415 mg/m^2 ^every 6 hours for 96 hours in 4-week cycles. Dose-limiting toxicity was not reached, demonstrating that high doses of lovastatin in this schedule are well tolerated and that bioactivity levels can be achieved [344]. Lovastatin as single agent has also been studied in advanced head and neck squamous cell carcinoma and cervical carcinoma. Maximum tolerated dosage (MTD) was determined as 7.5 mg/kg/day × 21 days every 28 days, and relevant plasma lovastatin levels were obtained. Although no objective responses were observed, median survival of patients in the study was 7.5 months, and stable disease for > 3 months was observed in 23% of patients. Interestingly, one patient achieved stable disease and clinical benefit for 14 months ON the study and a further 23 months OFF treatment [345]. The strongest evidence from the antitumor effects of statins was provided by Kawata et al., who performed a small randomized trial in 91 patients with unresectable hepatocellular carcinoma. Patients underwent transcatheter arterial embolization followed by oral 5-FU at 200 mg/day for 2 months. Patients were then randomly assigned to control (*n *= 42) and pravastatin (*n *= 41) groups at a daily dose of 40 mg. Median survival was 18 months in the pravastatin group vs. 9 months in controls (*p *= 0.006). The Cox proportional hazards model showed that pravastatin was a significant contributing factor to survival [346]. In general in all trials, statins were well tolerated and the main toxicity observed was rhabdomyolysis (muscle wasting), resolved with discontinued use and ubiquinone supplementation. Further clinical studies are strongly needed, particularly in combination therapy with other biologicals and classical cytotoxics, due to their synergy, as shown in several studies [347-353].

### Antimalarials

#### Chloroquine as antimalarial

Chloroquine is a 9-aminoquinoline specifically synthesized for use as an antimalarial agent early in the 1930s. This drug was widely used for malaria treatment and eradication efforts, which faltered in the 1960s following the development of drug-resistant parasites. Since that time, no antimalarial regimen has contained malaria as successfully and cost effectively [354]. In addition to its use in the antimalarial arsenal, chloroquine has been utilized for treatment of autoimmune diseases such as rheumatoid arthritis, due to its ability to slow the progress of the disease as a result of its immunomodulatory properties. Rheumatologists have also used chloroquine for treating systemic/discoid lupus erythematosus and other connective tissue disorders [355].

Chloroquine is commonly administered by oral route and has a very high volume of distribution, because it diffuses into the body's adipose tissue and is a lysosomotropic agent, i.e., it accumulates preferentially in the lysosomes of cells in the body. The pKa for the quinoline nitrogen of chloroquine is 8.5, i.e., it is ~10% deprotonated at physiological pH as calculated by the Henderson-Hasselbalch equation. This decreases to ~0.2% at a lysosomal pH of 4.6. Because the deprotonated form is more membrane-permeable than the protonated form, this results in a quantitative trapping of the compound in lysosomes.

Chloroquine's lysosomotropic character is believed to account for much of its anti-malarial activity. Chloroquine binds to heme to form what is known as the FP-chloroquine complex; this complex is highly toxic to the cell and disrupts the membrane's parasite function. The action of the toxic FP-chloroquine and FP results in cell lysis and ultimately, parasite cell autodigestion [356].

Chloroquine possesses a well known toxicity profile established during > 50 years of use in humans, which demonstrates the safety of its acute administration and low incidence of adverse events during chronic administration for periods of up to a few years. The most serious toxic effect is a macular retinopathy, which depends on the cumulative rather than the daily dose. The first report of retinal toxicity attributed to this drug was published by Cambiaggi [357], who described the classic retinal pigment changes in a patient receiving chloroquine for systemic lupus erythematosus treatment. One year later, Hobbs established an unquestionable association between long-term chloroquine use and the development of retinal pathology [358]. This pathology associated with chronic chloroquine use most likely results from chloroquine's affinity for melanin-containing structures, which increases its accumulation in pigmented ocular structures at concentrations much greater than in any other tissue in the body, even long after its use is discontinued. Chloroquine also accumulates in lymphocytes and macrophages, which results in antiinflammatory properties by reducing secretion of proinflammatory cytokines, and in particular of TNFα in monocytes/macrophages, as well as an important decrease in TNFα-receptor surface expression in human monocytic cell lines [359-361].

#### Chloroquine as anticancer drug

The mechanisms behind the effects of chloroquine on cancer are currently being investigated. The best known effects (investigated in clinical and pre-clinical studies) include radiosensitizing effects through lysosome permeabilization and chemosensitizing effects by drug efflux pump-transporter inhibition [362].

Chloroquine's lysosomotropic properties are the most probable mechanisms for many of the drug's biological effects, including radiosensitization. Because of its weak base properties, chloroquine accumulates in several intracellular organelles such as the endosome, Golgi vesicles, and the lysosomes, leading to cell dysfunction of several of these organelles [363]. In addition to its lysosomotropic properties, chloroquine has the ability to modulate cancer-cell autophagia, depending on the experimental model. Autophagy is an ancient cell-survival pathway that allows cells to recoup ATP and essential building blocks for biosynthesis when they are nutrient-starved or hypoxia-exposed, the hallmarks of the tumor microenvironment. This pathway involves the formation of double-membraned vesicles, coined autophagosomes, which envelop bulk cellular material and/or organelles and that subsequently fuse with lysosomes that degrade their cargo. Autophagy has been suggested to play important roles in the chemoresistance of cancer to some therapeutic agents, which typically induce an apoptotic response. To the contrary, others have argued that autophagy induction by anticancer agents increases their overall killing power, enabling death by both classical apoptosis and autophagy [364].

It has been suggested that chloroquine can affect p53-dependent cell death by inhibiting autophagy [365-367]. While some reports have suggested that chloroquine stimulates cell death by blocking the fusion of autophagosomes with lysosomes [368-370], other studies have suggested that chloroquine inhibits a later stage of autophagy by blocking degradation of cargo delivered to the lysosome [365]. In a lymphomagenesis mouse model, chloroquine showed to induce lysosomal stress and provoked p53-dependent cell death, which that does not require caspase-mediated apoptosis [371]. Existing pre-clinical information of chloroquine's antitumor effects and the broad experience in the use of this drug led Sotelo and his group to launch a small clinical trial that examined the potential benefit of adding chloroquine to a treatment regimen consisting of radiation plus carmustine in patients with glioblastoma. Results of this randomized phase II trial show that patients in the experimental arm survived twice as long as patients treated with the conventional regimen [372]. These provocative clinical results, as well as the body of evidence showing the important role of autophagy in cancer treatment, deserve the development of antitumor therapies based on chloroquine-based autophagic pathway modulation or on targeting other steps in the pathway.

### Antihormonal agents

#### Mifepristone as abortive

Mifepristone is a progesterone receptor antagonist and abortive, but was originally investigated for its antiglucocorticoid effects as a potential treatment for Cushing syndrome. In the presence of progesterone, mifepristone acts as competitive receptor antagonist, but is a partial agonist with weak activity when is present alone. Since 2000, mifepristone (commercially available as *Mifeprex*) was FDA-approved as abortive in combination with misoprostol.

Mifepristone acts at the receptor level, binding strongly to progesterone and glucocorticoid receptors; its binding affinity for these receptors is approximately five and three times greater than progesterone and dexametasone, respectively. Mifepristone, like progesterone, enters the target cell and reaches its receptors; however, it operates differently from progesterone, producing conformational changes in the receptor. When progesterone occupies its receptor, the receptor undergoes a conformational change resulting in dissociation from heat shock proteins, translocation to the nucleus, and binding to progesterone responsive elements (PREs) within target-gene promoter regions. This binding leads to gene transcription and protein synthesis. Mifepristone antagonizes these effects by occupying the receptor without stimulating gene transcription [373,374].

The pharmacokinetics of mifepristone is characterized by rapid absorption; time to peak serum levels is approximately 1–2 hours. Peak concentration rises according to the mifepristone dose within the 2–25 mg-dosage range. However, at a higher dose of 100–800 mg, C_max _values do not differ significantly, this likely a result of saturation. Bioavailability has been reported as 69% after oral intake of 200 mg of mifepristone [375].

Mifepristone is protein-bound in ca 94–99%; binding is principally to α1-acid glycoprotein (AAG). Distribution volume in women is reduced as the result of saturable high-affinity binding to AAG; therefore, serum AAG levels appear to limit tissue availability and could exert an impact on the pharmacokinetics of mifepristone in humans [376]. Mifepristone metabolism is initiated by rapid demethylation and hydroxylation in humans. Mifepristone half-life is 4 hours in rats, 15 hours in monkeys, and 30 hours in humans. *In vitro *studies conducted with human liver microsomes have shown that CYP450 3A4 is largely responsible for oxidative metabolism. Therefore, although specific drug or food interactions with mifepristone have not been completely studied, it is possible that ketoconazole, itraconazole, erythromycin, and grapefruit juice may inhibit its metabolism and increase mifepristone serum levels. In addition, rifampicin, dexamethasone, phenytoin, and phenobarbital may induce mifepristone metabolism and lower mifepristone serum levels. Demethylated and hydroxylated metabolites are excreted into bile, and in humans, only a small fraction of mifepristone can be detected in urine [377,378].

#### Mifepristone as anticancer agent

A number of studies have established that mifepristone could effectively inhibit PR-positive breast cancer proliferation [379,380], ovarian cancer [381-383], endometrial cancer [384], prostate cancer [385] and gastric cancer [386]. Despite several reports demonstrating that mifepristone can inhibit human cell growth, only limited information is available on the basic mechanism of this effect. Some *in-vitro *and *in-vivo *mechanisms involved in mifepristone antiproliferative effects in breast cancer show that mifepristone induces growth arrest and cell death, stimulating caspase-3, -8, and -9 activation in antiestrogen-resistant breast cancer cells [387]. It is known that traditionally, caspase-8 activation is used as an activation indicator of the extrinsic apoptosis pathway, whereas caspase-9 activation indicates involvement of the intrinsic mitochondrial apoptosis pathway [388].

In the case of endometrial cell-proliferation regulation, mifepristone is suggested as possessing an antioxidant mechanism [389]. Apoptosis induction has been reported by means of regulation of NF-kB [390], one of the early response transcription factors that play an important role in the regulation of genes involved in apoptosis. NF-kB up-regulation in endothelial cells stimulates apoptosis by 75%. Simultaneously with a marked increase in NF-kB activity, there is over-expression of the pro-apoptotic protein Bax, and down-regulation of the anti-apoptotic protein Bcl-2. It is also known that mifepristone can down-modulate the over-expression of two proteins involved in drug resistance, such as P-glycoprotein (P-gp) and Multidrug resistant protein (MRP) in lung cancer GLC4-Sb30 cells [391] and in human gastric adenocarcinoma SGC-7901 cells [392]. Mifepristone also induced apoptosis in human prostate cancer LNCaP cells by regulating Bcl-2 gene and TGFβ_1 _protein expression [385]. A cytostatic effect of mifepristone has also been shown in ovarian cancer cells by blocking DNA synthesis and cell cycle arrest at the G_1_-S transition via reduction of cdk2 activity, likely due to increased cdk2 association with cdk inhibitors p21 and -27 and reduced nuclear cdk2/cyclin E complex availability [383].

Another interesting mechanism described for mifepristone comprises its ability to modulate the activity of antitumor compounds such as doxorubicin and vinka alkaloids. There is evidence that some endogenous compounds as steroid hormones interact with P-gp [393], and corticosteroids and mineralocorticoids are also P-gp transport substrates [394]. Moreover, some steroid antagonists, such as tamoxifen and toremifen, interfere with P-gp function by virtue of their hydrophobicity and the presence of phenyl rings [395], structural characteristics shared by mifepristone. Thus, it has been reported that mifepristone enhances doxorubicin cellular accumulation in resistant human leukemia K562 cells and RHCL rat hepatoma cells [396], suggesting an inhibitory effect on P-gp function, a mechanism of action demonstrated for other chemosensitizer agents including verapamil and cyclosporine. Recently, it has been shown that mifepristone enhances cisplatin chemosensitivity in resistant ovarian cancer cell line [397], **a **finding consistent with the data of Liu et al. [398], demonstrating, in a mouse model bearing xenografted cisplatin-resistant ovarian carcinoma, significantly greater inhibition rates of tumors in the combined treatment in comparison with cisplatin treatment alone.

Data have recently been reported on mifepristone participation in modulation of the cisplatin effect in human cervical cancer cell lines (negative estrogen [ER-] and progesterone [PR-] receptors). Cisplatin's antiproliferative effect was potentiated in combination with mifepristone (10 μM). The results also showed that intracellular cisplatin levels increased approximately 2-fold due to mifepristone pre-treatment. The mifepristone dose employed in the previously mentioned work is within the plasma concentration range usually observed in humans after oral administration of the drug [399].

The effect of mifepristone has also been widely studied in meningioma, which is often progesterone – but not estrogen – receptor-positive. In this model, mifepristone elicits potent growth-inhibitory effects *in vitro *and in human xenografts. Interestingly, this agent is also active regardless of the presence of the PR in meningioma cells, suggesting off-target effects that contribute to their antitumor activity [400,401].

Clinical studies of mifepristone have demonstrated its anti-meningioma activity. In a study from The Netherlands, 10 patients with recurrent or primary inoperable meningiomas, all of whom had shown recent neuroradiological and/or ophthalmological evidence of tumor growth, were treated with 200 mg mifepristone daily for 12 months. There were three patients with stable disease and regression in three patients, as well as a decrease in complaints of headache, and improved general well-being was observed in five patients. Two patients died during the treatment period from unrelated causes [402]. In a larger study, 28 patients received daily oral mifepristone at 200 mg/day for a total of 1,626 patient months-of-treatment. Patients also received oral dexamethasone 1 mg/day for the first 14 days of treatment. At a median therapy duration of 35 months (range, 2–157 months), mifepristone was well tolerated with mild fatigue (22 patients), hot flashes (13 patients), and gynecomastia/breast tenderness (six patients), these three the most common side effects. However, endometrial hyperplasia or polyps were documented in three patients, and one patient developed peritoneal adenocarcinoma after 9 years of therapy. In another study, minor responses (improved automated visual field examination or improved Computer tomography or Magnetic resonance imaging scan were noted in eight patients, seven of whom were males or pre-menopausal females. Authors agree on the feasibility and tolerability of this treatment and on that even minor regressions can result in significant clinical benefit [403].

A phase II study of mifepristone in cisplatin-resistant ovarian cancer was reported in 44 patients who received 200 mg orally on a daily basis. Among response-evaluable 34 patients, overall response rate was 26.5% (nine patients); of these, three (9%) had complete response, and six (17.5%), partial. The response of one patient in each group was measured by CA-125 levels, while the remainder of patients had measurable disease. Responses lasted 1–4 months in all but one patient and one patient, who continued in response for > 3 years. The major toxic effect was a rash [382]. Mifepristone appears to possess activity against recurrent uterine leiomyosarcomas. A dramatic response lasting > 3 years was observed in one case of three patients with recurrent low-grade progesterone receptor-positive leiomyosarcoma [404].

#### Conclusions and perspectives

Drug-development strategies against cancer are changing. After nearly 50 years of using cytotoxics, current anticancer drugs – approved and in development – were sought based on target-driven approaches, thus the name targeted drugs. These drugs are regarded as specific, which commonly means that they are aimed at hitting one or a few key cellular targets. Agents that target single signaling molecules often exhibit limited clinical activities, at least in the major solid-tumor groups, with the exception of certain well known examples represented by imatinib in chronic granulocytic leukemia and gastrointestinal stromal tumors, lending support to the gene addiction theory [405]. This is not surprising, because agents that affect a single target (single-hit agents) might not always affect complex systems in the desired manner even if they change the behavior of their immediate target completely. For example, single targets might have back-up systems that are sometimes sufficiently different in not responding to the same drug, and many cellular networks are robust and prevent major changes in their outputs despite dramatic changes in their constituents. These considerations are independent of whether or not the pharmacological agent inhibits or activates its target [406]. Paradoxically, in drug development, it is common that poor specificity in a drug is perceived as a negative characteristic. However, this paradigm is changing, and the possibility of exploiting promiscuity is considered in novel approaches for treating complex disorders such as cancer, depression, and cardiovascular disease [407-409]. Complex disorders ultimately share the same underlying pathological processes that play a particularly prominent role in their etiology. These common mechanisms comprise, among others, inflammation, angiogenesis, fibrosis, cellular proliferation, and defective apoptosis. Although inflammation is a defense mechanism, an inappropriate inflammatory response is the cause of many diseases, including cancer, multiple sclerosis, inflammatory bowel disease, rheumatoid arthritis, endometriosis, arteriosclerosis, and psoriasis. Therefore, an understanding of the cellular effectors and mediators that play key roles in the different inflammatory diseases can guide the ultimate positioning of anti-inflammatory agents. Similarly, angiogenesis is a key disease process in multiple indications, including tumor development and metastasis, age-related macular disease, arthritis, endometriosis, and psoriasis. Fibrosis is another common mechanism that encompasses diseases with prominent fibrotic etiology, such as lung fibrosis, liver cirrhosis, renal failure, and tissue scarring. Because fibrotic processes are often downstream of inflammatory processes, inflammation and fibrosis may represent optimal drug targets for the same disease at different clinical disease-progression stages. More than one common mechanism may have an impact on a particular disease, as observed in arthritis, endometriosis, and psoriasis, and this may provide opportunities for combination therapies with synergistic effects [410-412].

Simultaneous modulation of multiple targets is often required to alter a clinical phenotype of robust systems such as the molecular networks of living cells. Biological redundancies and alternative pathways can often bypass the inhibition of a single target or of multiple targets along a single pathway, suggesting that in some cases, broad-specificity compounds or multitarget drug therapies may be more effective than individual high-affinity, high-specificity therapies. Multiple but partial attacks mimic a number of *in vivo *scenarios well and may be useful in the efficient modification of other complex systems [413]. Noteworthy examples of these concepts are the relative success of drugs inhibiting multiple kinases and/or display off-target activities in the treatment of previously difficult-to-treat diseases such as renal and liver carcinoma, as well as cancer drugs that instead of hitting a single gene product, hit processes such as proteosome, heat shock protein 90 (Hsp90), and HDAC inhibitors [414].

With all this experimental and clinical evidence in the cancer field, it comes as no surprise that a number of widely used drugs for conditions other than cancer hit the same primary and/or secondary targets of known anticancer drugs developed as such. Contrariwise, drugs developed as anticancer agents are used for benign conditions, such as paclitaxel and bevacizumab for local catheter-based, antiproliferative-drug delivery for prevention of coronary restenosis and ocular neurovascular disease, respectively [415,416]. Others, such as those whose off-target effects on DNA methylation machinery led to thinking of them as anticancer agents remain to be studied. For instance, the Na+ channels are the primary target of procainamide and procaine as antiarrhythmics and local anesthetics, however, Na+ channels have recently found to participate in cancer development [417]. Among all these agents, perhaps the most remarkable is metformin, which on targeting AMPK could be at least as – if not more – effective than mTOR, Fatty acid synthase, and mevalonate pathway inhibitors.

After all, cells work through signaling pathways [418]. Defining the role of pathways in complex diseases will undoubtedly take many years. Perhaps, future textbooks instead of being organized by organ and systems, pathology, or physiology, will be categorized by the signaling pathways involved. In this escenario, drugs would not be denominated anticancer or antidiabetic or antihypertensive; they would be named by the signaling pathway they inhibit.

In the meanwhile, public and not-profit organizations must be encouraged to rapidly translate the pre-clinical data of known non-cancer drugs into phase II and III clinical trials, as well as to conduct more research on the potential cancer activities of this type of drugs. In the long run, these strategies may motivate changes that may aid in cancer drug availability to a growing and underserved population worldwide.

In 1881, the Mark Twain novel *The Prince and the Pauper *was published. Set in 1547, the novel relates the story of two young boys who are identical in appearance but who live under opposite social circumstances, which render them unable to function in the world that is as familiar to one as to the other. In many ways, the book is a social satire, particularly compelling in its condemnation of the inequality that existed among the classes in Tudor England. The significance of the novel is quite contemporaneous with the current perception by researchers and oncologists regarding cancer drugs. All of these – Oncologists, cancer researchers, and patients alike, and, why not, biomedical journal editors with strong ties with the pharmaceutical industry – must be aware that is not just princely (read expensive) drugs that can help to treat cancer, but that pauper (read inexpensive) drugs are being developed and bear the same potential for efficacy. If these drugs eventually possess the latter, they should not be regarded as pauper drugs solely because they are not advertised by Big Pharma.

## Competing interests

The authors declare that they have no competing interests.

## Authors' contributions

AD-G conceived and wrote the manuscript. PGL, LAH, AG-F and MC drafted parts of the manuscript. JL MF critically read the manuscript for important intellectual content. All authors read and approved the final manuscript.
